# A functionally divergent intrinsically disordered region underlying the conservation of stochastic signaling

**DOI:** 10.1371/journal.pgen.1009629

**Published:** 2021-09-10

**Authors:** Ian S. Hsu, Bob Strome, Emma Lash, Nicole Robbins, Leah E. Cowen, Alan M. Moses

**Affiliations:** 1 Department of Cell & Systems Biology, University of Toronto, Toronto, Canada; 2 Department of Molecular Genetics, University of Toronto, Toronto, Canada; 3 Department of Computer Science, University of Toronto, Toronto, Canada; University of Rochester, UNITED STATES

## Abstract

Stochastic signaling dynamics expand living cells’ information processing capabilities. An increasing number of studies report that regulators encode information in their pulsatile dynamics. The evolutionary mechanisms that lead to complex signaling dynamics remain uncharacterized, perhaps because key interactions of signaling proteins are encoded in intrinsically disordered regions (IDRs), whose evolution is difficult to analyze. Here we focused on the IDR that controls the stochastic pulsing dynamics of Crz1, a transcription factor in fungi downstream of the widely conserved calcium signaling pathway. We find that Crz1 IDRs from anciently diverged fungi can all respond transiently to calcium stress; however, only Crz1 IDRs from the Saccharomyces clade support pulsatility, encode extra information, and rescue fitness in competition assays, while the Crz1 IDRs from distantly related fungi do none of the three. On the other hand, we find that Crz1 pulsing is conserved in the distantly related fungi, consistent with the evolutionary model of stabilizing selection on the signaling phenotype. Further, we show that a calcineurin docking site in a specific part of the IDRs appears to be sufficient for pulsing and show evidence for a beneficial increase in the relative calcineurin affinity of this docking site. We propose that evolutionary flexibility of functionally divergent IDRs underlies the conservation of stochastic signaling by stabilizing selection.

## Author summary

During the last decade or so, it has been appreciated that transcription factors sometimes control gene expression by entering the nucleus in short, stochastic “pulses”, as opposed to (seemingly simpler) stable nuclear localization. The amino acid sequences that encode this type of dynamic regulation are found in so-called intrinsically disordered regions that do not fold into stable 3D conformations. Thusfar, little is known about the evolution of transcription factor pulsing. Here we studied the rapidly evolving intrinsically disordered reigons underlying pulsing of the yeast transcription factor, Crz1. We find that the intrinsically disordered regions are highly diverged in their amino acid sequences, but for some species they can still perform the pulsing function when expressed in yeast, while for more distantly related species they can not. Surprisingly, for these distantly related species, we found that Crz1 does pulse in the cells of the distantly related species. We propose that there has been compensatory evolution to preserve pulsing, even though the intrinsically disordered regions have diverged. This type of evolutionary preservation is called “stabilizing selection,” and we believe that it is working to preserve the functional output of signaling pathways (in this case pulsing) even when the protein components (or at least their intrinsically disordered regions) are changing.

## Introduction

One of the most remarkable features of living cells is their ability to transmit and process information about their surroundings. It is now appreciated that the dynamics of molecules connected in regulatory networks and signaling pathways underlie many of these capabilities [1–3]. But how do peptide sequences underlying this ability evolve? Relative to enzymatic functions whose evolution has been studied for decades [4–7], research on the evolution of cellular information transmission and signal processing systems is only beginning to emerge [8]. Most research on signaling evolution has been focused on the specificity of kinases, receptors, and transcription factors [8], and, to our knowledge, a comparison across species of p53 [9] and Msn2/4 dynamics (Dr. Yihan Lin, personal communication) are the two lone evolutionary studies of stochastic signaling dynamics. Although evolutionary rewiring of DNA-protein interactions in cis-regulatory networks has been described [10], the evolution of the molecular mechanisms that lead to post-translationally controlled signaling dynamics is much less characterized [11–14]. Part of the difficulty in obtaining a mechanistic understanding of signaling evolution is that post-translational regulation and transient signaling interactions are often encoded within rapidly evolving IDRs [15–17], which are largely refractory to ancestral protein reconstruction approaches [8,18].

Here we consider the evolution of the IDRs controlling the pulsatile dynamics of Crz1, a transcription factor in budding yeast that responds to rapid fluctuations of cytosolic calcium concentration (which we refer to as calcium bursts [19]). Pulsatile dynamics are steady state stochastic fluctuations that encode information via frequency modulation of kinase activity (e.g., ERK [20]), protein abundance (e.g., p53[21]), and cytoplasm-to-nuclear-translocalization (e.g., Msn2/4 [22], NFATC1[23,24]). Crz1 has been found to control gene expression through the frequency modulation of post-translationally controlled nuclear-localization pulses [25]. A recent study has also found that Crz1 pulsing and continuous Crz1 nuclear localization are differently decoded by promoters, providing a causal connection between pulsing dynamics and differential gene expression [26]. However, to our knowledge, in no case has the fitness benefit of pulsatile dynamics been established, nor has a mechanism for their evolution been proposed, save for one pioneering study of Msn2/4 pulsing (Dr. Yihan Lin, personal communication).

**Fig 1:**
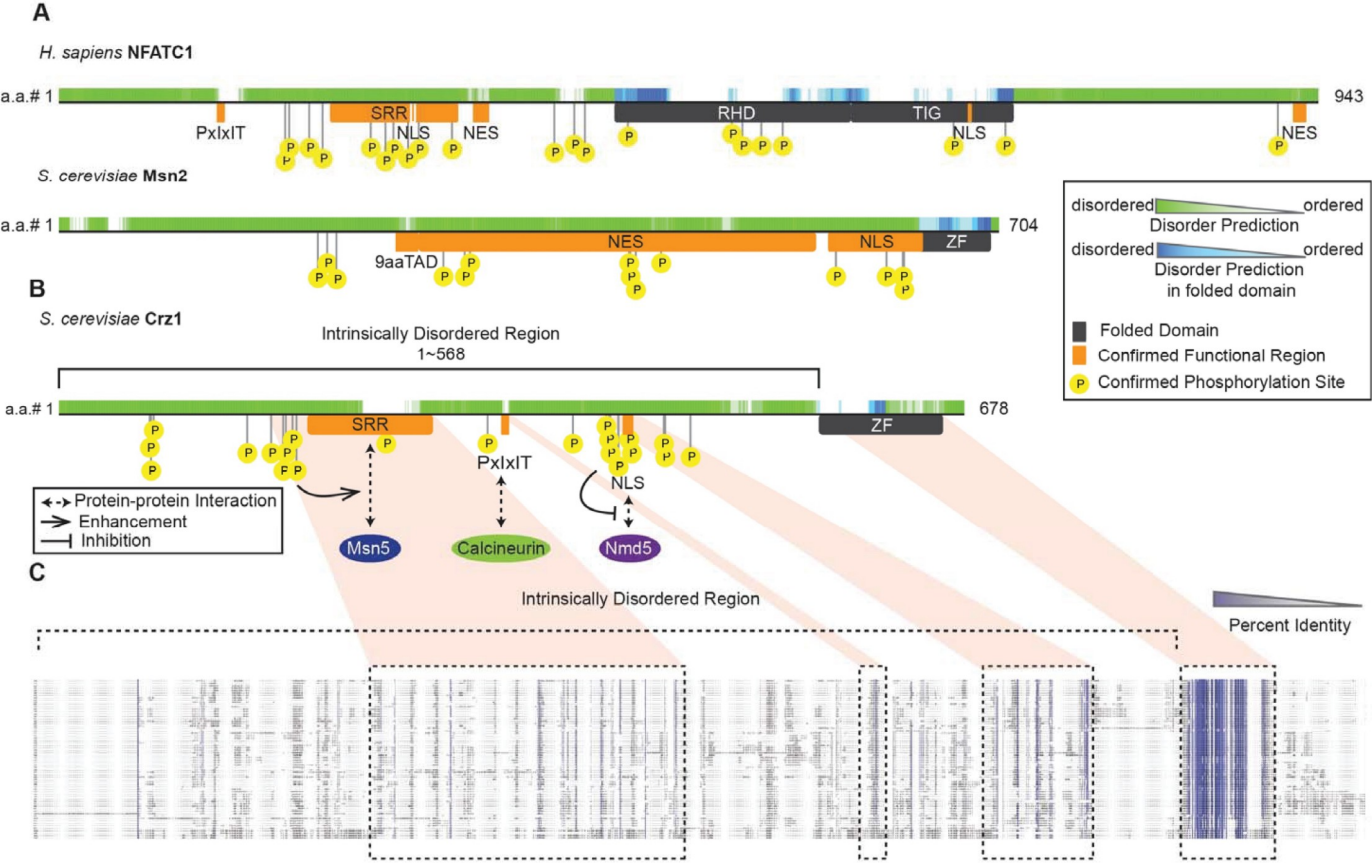
The IDRs of Crz1 and other pulsatile transcription factors contain functional elements involved in the mechanism of nuclear-cytoplasmic translocation. A, B) Schematic representations of three pulsatile transcription factors (NFATC1 and Msn2 in A), Crz1 in B)). The upper half of each representation shows predicted disorder (D2P2, [81]), and the lower half of each representation shows the known functional regions [82,83]. SRR: serine-rich region. PxIxIT: *S*. *cerevisiae* calcineurin docking site PxIxIT. NLS: nuclear localization signal. RHD: Rel homology domain. TIG: transcription factor immunoglobin. 9aaTAD: nine-amino-acid transactivation domain. ZF: zinc finger. B) A schematic drawing of the protein-motif interactions on the protein sequence of Crz1 from *S*. *cerevisiae*. C) A schematic representation of the sequence alignment of the Saccharomyces fungi from the Yeast Gene Order Browser (YGOB, [84]). Purple shades represent the percent identity of each position [85]. Boxes represent the homologous regions of the elements on Crz1 from *S*. *cerevisiae*. Orange shadows link the corresponding regions between B) and C).

Crz1 is widely conserved in fungi [27,28], but, although the subcellular localization of Crz1 orthologues has been studied in the distantly related fungi *Schizosaccharomyces pombe* [29,30], *Candida albicans* [31,32], and *Cryptococcus neoformans* [33,34], no evidence for pulsing has been reported. Whether Crz1 pulsing is conserved over evolution or whether it is beneficial to the cells has, to our knowledge, not been established. Like other stochastically pulsatile transcription factors, e.g., Msn2 [35] and NFATC1 [36] (Fig 1A), Crz1 contains more than 500 amino acids that are predicted to be intrinsically disordered (Fig 1B, [37]) and contain numerous interaction and post-translational modification sites (e.g., calcineurin docking site, nuclear export signal (NES), nuclear localization signal (NLS), Fig 1B, [38–41]). As expected for an intrinsically disordered region [42], this region shows little primary sequence similarity (Fig 1C).

Rapidly evolving IDRs have been shown to support similar functions when expressed in *S*. *cerevisiae* [43]. Those observations led to the model that functional output of the orthologous IDRs was maintained because a molecular feature (basal net charge) was preserved by stabilizing selection. Subsequent work showed that preserved molecular features could be used to predict functions of IDRs and help construct mechanistic hypotheses [44]. However, it is unknown which molecular features in the Crz1 IDR are necessary for pulsing, or whether these features are preserved among the highly diverged IDRs found in Crz1 orthologues in other fungi.

In this study, we show that, when expressed in *S*. *cerevisiae*, two orthologous Crz1 IDRs from the Saccharomyces clade support pulsatility, transmit environmental information, and rescue fitness in growth competition assays, while two orthologous IDRs from more distantly related fungi do none of the three. Despite the differences observed when expressed in *S*. *cerevisiae*, we show that Crz1 orthologues pulse in the native systems of the two distantly related fungi, consistent with conservation of the pulsing phenotype over long evolutionary time. The conservation of phenotype but lack of conservation of IDR function indicates that, within the disordered region, evolutionary changes in some elements needed for complex signaling dynamics have compensated for evolutionary changes in others. This pattern of compensatory evolution in the context of preserved function is a hallmark of stabilizing selection [45]. By comparing IDR sequences of Crz1 orthologues, we infer that one of these evolutionary changes is in the calcineurin docking site, PxIxIT, which increased binding strength during evolution. Remarkably, by experimentally increasing the PxIxIT strength in a distantly related IDR to the Saccharomyces PxIxIT strength (via three point-mutations), we can rescue pulsing and improve fitness in a competition assay. Our study demonstrates that stochastic pulsatility is beneficial and that a position-dependent molecular feature in the IDR plays a role in rewiring the molecular basis of the stochastic signaling pathway, even though the phenotype is preserved.

## Results

### Evolutionary changes in Crz1 IDRs are associated with changes in Crz1 pulsing

We noted large intrinsically disordered regions in a few well-studied pulsing transcription factors (Fig 1). Therefore, we asked if pulsing transcription factors contain larger disordered regions than expected based on all transcription factors in *S*. *cerevisiae*. We found that 6 of 9 pulsing TFs in yeast have more than 60% of their sequence predicted to be disordered (see Methods), which is more than expected for all transcription factors (41/226, P<0.05, Fisher’s test, S1 Fig). We then sought to confirm that the IDR of Crz1 was responsible for pulsing. To do so, we designed a passive reporter system in *S*. *cerevisiae* that expresses an IDR tagged with GFP. We found that the *S*. *cerevisiae* disordered region alone showed pulsing with similar dynamics as the endogenous protein, although the expression level of the protein was lower (S2 Fig). Therefore, we fused a defective Crz1 DNA-binding domain [46] tagged with GFP to the disordered regions (denoted as Sc-reporter) and found nearly endogenous dynamics and expression levels, indicating that the disordered region is sufficient for the pulsing dynamics but that the DNA binding domain is needed for protein stability (see methods for more details).

Since the calcium/calcineurin signaling pathway is highly conserved [27], we predicted that functional elements within the disordered regions would be conserved over evolution if the dynamics of Crz1 are important for signaling function [25]. Consistent with this, some functional elements important for the control of subcellular localization, such as the nuclear localization signal (NLS), nuclear export signal (NES), and the calcineurin docking site PxIxIT [38,39] (summarized in Fig 1B), are found in orthologous Crz1 sequences. On the other hand, overall, the IDRs of Crz1 are highly diverged (little sequence similarity is detected in sequence alignments, Fig 1C), which leads to an opposite prediction that the dynamics of Crz1 orthologues would diverge as has been found for p53 [9]. To quantify Crz1 dynamics in response to the upstream calcium signaling pathway, alongside the GFP-tagged Crz1 IDR reporter (denoted as “pulsing reporter”), we expressed a calcium sensor GCaMP3 [47,48] in a “double-reporter strain” (see methods for more details).

**Fig 2:**
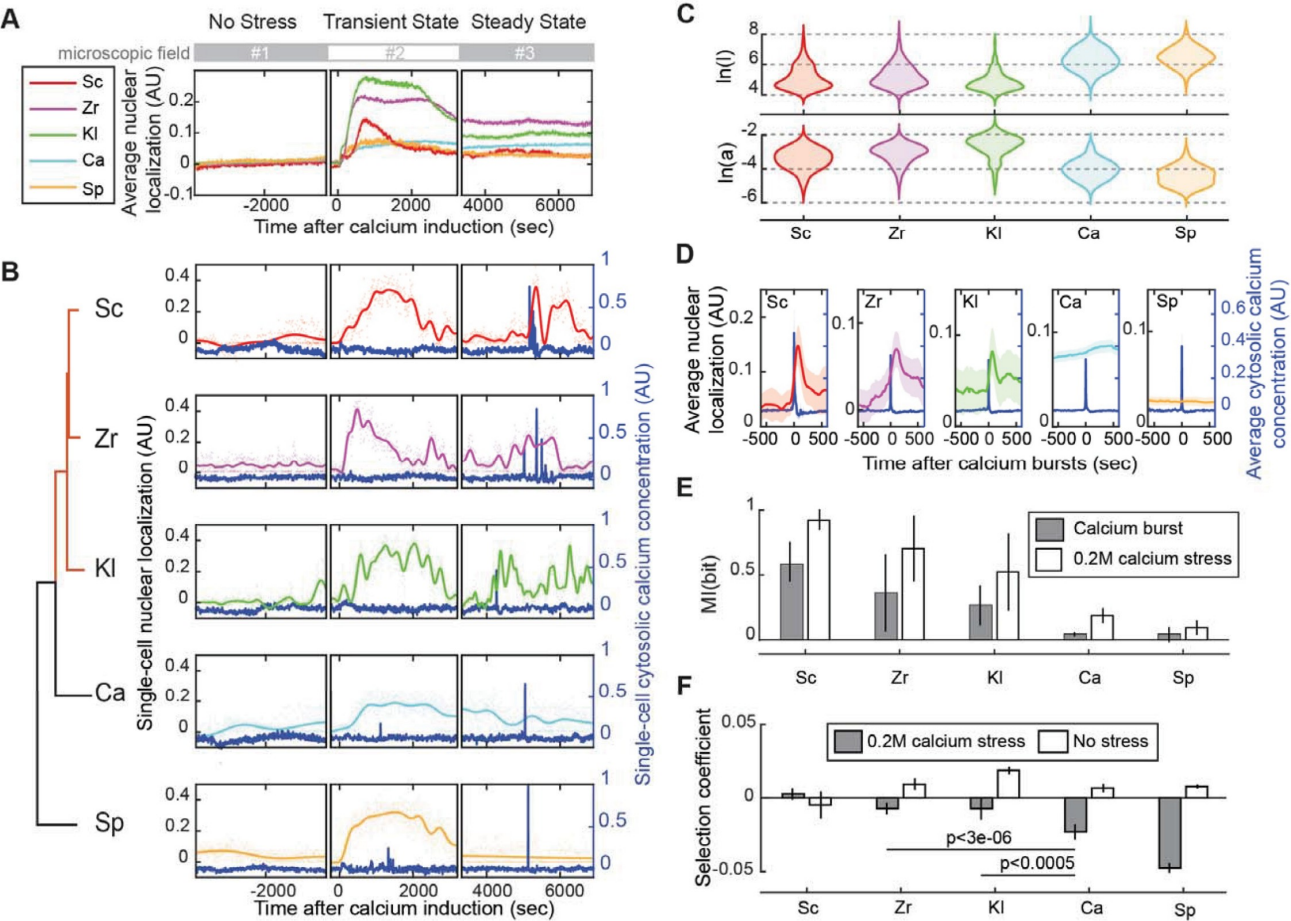
The IDRs of Saccharomyces species rescue both pulsing phenotype and fitness under 0.2 M calcium stress. A) Population averaged localization traces show nuclear localization in response to 0.2 M calcium stress in every reporter strain, corresponding to the Crz1 IDR from the species indicated (Sc, Zr, Kl, Ca, Sp, indicate *S*. *cerevisiae*, *Z*. *rouxii*, *K*. *lactis*, *C*. *albicans*, and *S*. *pombe*, respectively). n > 100 cells for each strain. Shadow areas indicate 1.96 SE. Plots are broken to indicate when each experiment switched to a different microscope field to avoid laser-induced nuclear localization. B) Representative single-cell trajectories of cytosolic calcium concentration (blue lines) and nuclear localization (color-coded lines), estimated by Gaussian Process regression on 600 time-points (color-coded dots). Cytosolic calcium concentration is quantified as cytosolic GFP intensity from the calcium sensor GCaMP3. Plots are broken to indicate when each trajectory composites a different cell from a different microscope field. The reporter strains (indicated by species names as in A) are ordered according to a phylogenetic tree showing the Saccharomyces clade (red) and the outgroup (black). C) The distribution of dynamic parameters estimated from single-cell time-lapse data. *a* determines the average distance of the trajectory away from its mean, and *l* determines the length of the fluctuation on the trajectory. D) Pulse-triggered averaging of the trajectories of pulsing reporters (black lines) around calcium bursts (blue lines). Time is relative to calcium bursts. Shadow areas indicate 1.96 SE. n > 100 bursts for each strain. E) Averaged mutual information between calcium bursts and dynamics of pulsing reporters (grey bars) and between 0.2M stress and GP parameter values (white bars). n = 1, 3, 3, 3, 1 replicate for Sc, Zr, Kl, Ca, Sp, respectively. F) The selection coefficient measured under 0.2M calcium stress (grey bars) or no stress (white bars). Error bars represent 1.96 SE. P-values indicated are from a two-tail two-sample t-test. n > 10 replicates for each competition assay with at least three cell lines. AU: Arbitrary Units.

We first confirmed that, as expected based on previous reports for *S*. *cerevisiae*, *C*. *albicans*, and *S*. *pombe* [29–32], every reporter strain showed transient nuclear localization as a response to 0.2M calcium induction (Fig 2A). However, only Saccharomyces reporters (*S*. *cerevisiae* (Sc), *Zygosaccharomyces rouxii* (Zr), and *Kluyveromyces lactis* (Kl)) showed pulsing dynamics during steady state (Fig 2B, more examples in S3 Fig). The two distantly related reporters (*C*. *albicans* (Ca) and *S*. *pombe* (Sp)) transiently localized to the nucleus after the calcium induction and then continued with stable nuclear localization during steady state (Fig 2B, more examples in S3 Fig). To quantify these phenotypic differences at the single-cell level, we measured the duration and amplitude of reporter dynamics by fitting Gaussian Processes to the single-cell trajectories [49]. The results suggest that, compared to the outgroup, Saccharomyces reporters have a shorter duration (represented by low ln(l)) and higher amplitude (represented by high ln(a)) in their dynamics (Fig 2C). Because Crz1 pulses are known to follow calcium bursts [19], we adapted the technique of pulse-triggered averaging [50] to investigate the average dynamics of pulsing reporters around calcium bursts. Consistent with the pulsatile dynamics observed in single-cell trajectories, Saccharomyces reporters quickly responded to calcium bursts on average (Fig 2D). In contrast, the average dynamics of the outgroup reporters are not affected by calcium bursts (Fig 2D).

Next, we applied two ways to estimate the amount of information encoded in the dynamics of pulsing reporters. Information-theoretic approaches provide a natural framework to estimate the information transmission capacity of cellular signaling pathways [51,52]. Because cells show calcium bursts during steady state (where cells still experience calcium stress but have adapted to it), we estimated the mutual information between the dynamics of cytosolic calcium concentration and pulsing reporters and defined it as “calcium burst information” (Fig 2E grey bars). To do so, we categorized each time point of a single-cell calcium trajectory into “calcium burst” and “not calcium burst” and estimated how much information about the presence of calcium bursts is encoded in a short period of the Crz1 pulsing dynamics after the calcium bursts (see Methods for details). We found that the dynamics of the Saccharomyces reporters encoded more mutual information than that of the distantly related reporters (mean MI = 0.41 bits vs. 0.04 bits, 2-tails t-test, p = 0.004, n = 9 and 6, respectively). We also estimated mutual information between the presence or absence of 0.2M external calcium stress and the dynamics of pulsing reporters and defined it as “calcium stress information” (Fig 2E white bars). In other words, we estimated how much information about the presence of external stress is encoded in the Crz1 pulsing dynamics of a population (see Methods for details). Again, we found more mutual information in the Saccharomyces reporters (mean MI = 0.72 bits vs. 0.13 bits, 2-tails t-test, p <10^−3^, n = 9 and 6, respectively). Taken together, these results suggest that pulsing dynamics encode additional information about the environment. Consistent with the model of stabilizing selection on molecular features [43,44], these results also suggest that some functional sequence properties were preserved in the Crz1 IDR of the Saccharomyces clade. On the other hand, the differences in function observed for the distantly-related Crz1 IDRs rules out the idea that all the molecular properties of these IDRs were preserved by stabilizing selection.

### IDRs that rescue pulsing also confer a fitness benefit in 0.2M calcium stress

Given that the IDRs from distantly-related fungi do not support pulsing in *S*. *cerevisiae*, we next sought to test whether these IDRs support cell growth. Since all the orthologous IDRs support calcium-induced transient nuclear localization (Fig 2A and 2B, [29,32]) and contain consensus calcineurin docking sites [53], a serine-rich NES region [29–32], and several conserved phosphorylation sites [12] (Fig 1C), we wondered if these are sufficient for cell fitness. Therefore, we sought to directly measure cell fitness under calcium stress in a competition assay (see Methods). To confirm that Crz1 function is needed for fitness in our assay conditions, we tested a mutant with *CRZ1* deletion as well as a mutant in which conserved phosphorylation sites in the serine-rich NES region are removed (“mSRR” [39]). As expected, we found a large fitness defect for the *CRZ1* deletion, and a small but significant fitness defect for mSRR strain, confirming that our assay has the power to detect both large and small effects on fitness (S4 Fig). The fitness defect of the mSRR strain suggests that the dynamics of Crz1 [2] are important for fitness (and not the average level of nuclear localization) because it responds to calcium stress by moving to the nucleus but does not pulse (S4 Fig).

We next compared the fitness of strains where the endogenous IDRs of Crz1 were replaced with orthologous sequences. Consistent with the idea that pulsing is beneficial to the cell, we found that IDRs from the Saccharomyces clade, but not from the outgroups, rescued fitness in this assay (Fig 2F). The observed fitness defects are conditional on the 0.2M calcium stress (Fig 2F) and can be observed on the time-scale of our time-lapse microscopy experiments (S5 Fig), consistent with the known functions of Crz1[25] and ruling out misfolding or misexpression effects. Although the IDRs of both distantly related species *C*. *albicans* and *S*. *pombe* support transient nuclear localization after calcium exposure (Fig 2A), mutants with the outgroup IDRs showed fitness defects comparable to the phosphorylation site mutants (S4 Fig), again indicating that constant nuclear localization in response to calcium stress is not sufficient for full fitness. In addition, since the outgroup IDRs show an average steady-state nuclear localization similar to that of the wild type IDR (Fig 2A), but different steady-state dynamics (Fig 2B), the difference in stress survival between mutants with pulsing and non-pulsing IDRs is not due to the average steady-state Crz1 nuclear localization level. Together, these results are consistent with the idea that pulsing transmits important extra information when cells are under calcium stress that is beneficial for cell growth, but we note that we cannot rule out other consequences of the changes in IDR sequences whose effects we have not measured.

### Pulsing is conserved, but the underlying mechanisms have changed

**Fig 3:**
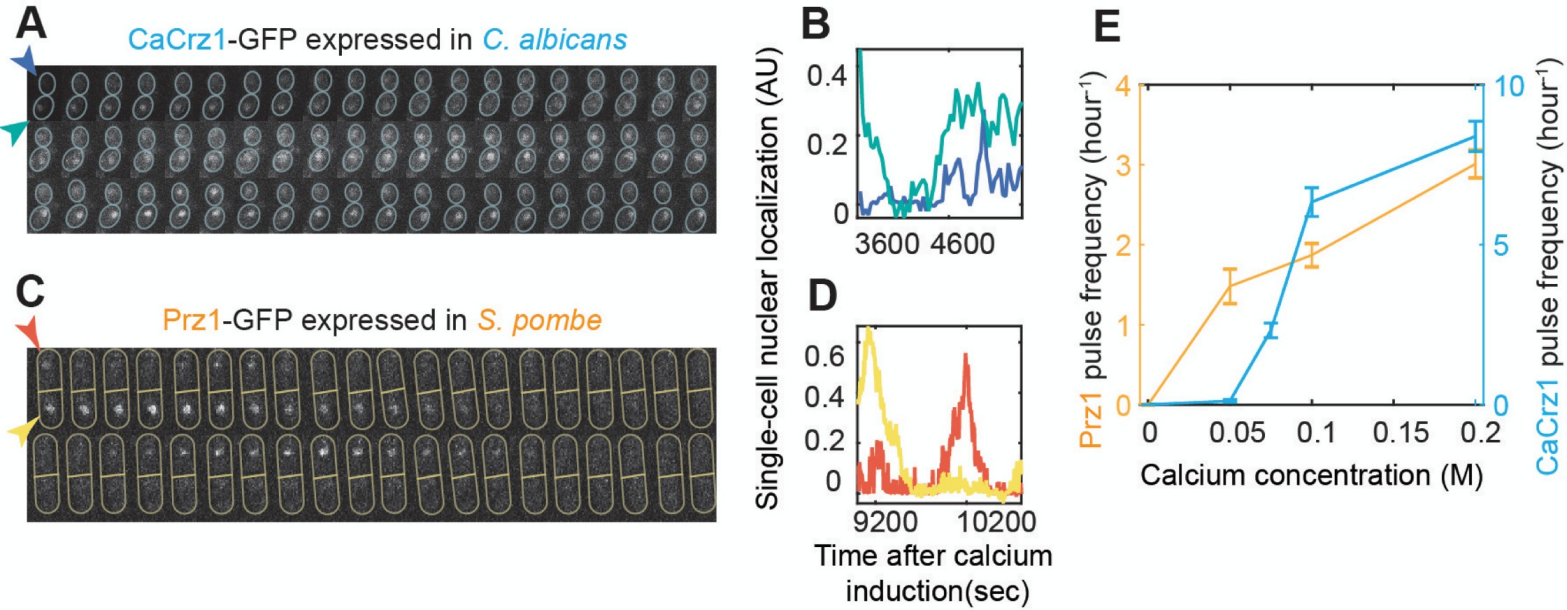
*C*. *albicans* and *S*. *pombe* show pulsing and frequency modulation. Filmstrips are showing *C*. *albicans* (A) and *S*. *pombe* (C) with GFP-tagged Crz1 orthologues (CaCrz1 and Prz1, respectively) during steady state after the addition of 0.2 M extracellular calcium. Yeast cells are outlined in each frame and indicated through the arrows in the first frame, which are colored accordingly to the single-cell trajectories in B) and D). A) Frames are separated by 30 seconds, and the actual time resolution in B) is the same. C) Frames are separated by 45 seconds, but the actual time resolution in D) is 6 seconds per frame. Image acquisitions of each species were performed through different microscopes. E) Populational average of pulse frequency increases with calcium concentration for both Crz1 orthologues. Error bars represent 1.96 SE. n > 30 in each experiment. AU: Arbitrary Units.

We next asked if Crz1 pulsing can be found in the native systems of the distantly related fungi. Based on the results above and the lack of previous reports of pulsing in the other species [29–32], we hypothesized that pulsing evolved along the lineage leading to the Saccharomyces from a non-pulsing ancestor, and we did not expect the distantly related species to show Crz1 pulsing. On the other hand, if selection preserved the pulsing phenotype, we would expect to find pulsing in the other species. Under the model where selection has preserved pulsing, our finding that the IDRs from distantly related fungi do not pulse in *S*. *cerevisiae* implies that there must be compensatory changes that maintain pulsing in those species [45]. To distinguish between these models, we obtained strains ([30] and methods) of the two distantly related fungi (*C*. *albicans* and *S*. *pombe*) that express endogenous GFP-tagged Crz1 orthologues (CaCrz1 and Prz1) and measured pulsing under 0.2M calcium stress. Remarkably, both CaCrz1 (Fig 3A and 3B, S1 Movie, more examples in S6 Fig) and Prz1 (Fig 3C and 3D, S2 Movie, more examples in S6 Fig.) pulse in *C*. *albicans* and *S*. *pombe*, respectively, ruling out our hypothesis that pulsing evolved only along the lineage leading to the Saccharomyces.

Pulsing of Crz1 in *S*. *cerevisiae* shows frequency modulation [25], where the rate (per unit time) of pulsing increases with greater calcium stress. Therefore, we measured pulsing in the outgroup species at several calcium concentrations, and, consistent with conservation of frequency modulation, we also found a correlation between CaCrz1 and Prz1 pulse frequency and the strength of calcium stress (Fig 3E). We note that this observation also rules out the possibility that our observations of pulsing in these other strains are an artifact of microscopy or laser stress [54]. The results are consistent with the model that Crz1 pulsatility (and frequency modulation) has been conserved by natural selection for a long evolutionary time, which is also consistent with our observation of a fitness benefit for pulsing in *S*. *cerevisiae*. However, the lack of pulsing of more distantly related IDRs (and failure to rescue fitness under calcium stress) when expressed in *S*. *cerevisiae* implies that protein-protein interactions between the IDRs and the calcium signaling pathway have changed in some way, while the output of the regulatory network has remained the same.

### PxIxIT strength in a specific part of the Crz1 IDR increased in the Saccharomycetacea clade

**Fig 4:**
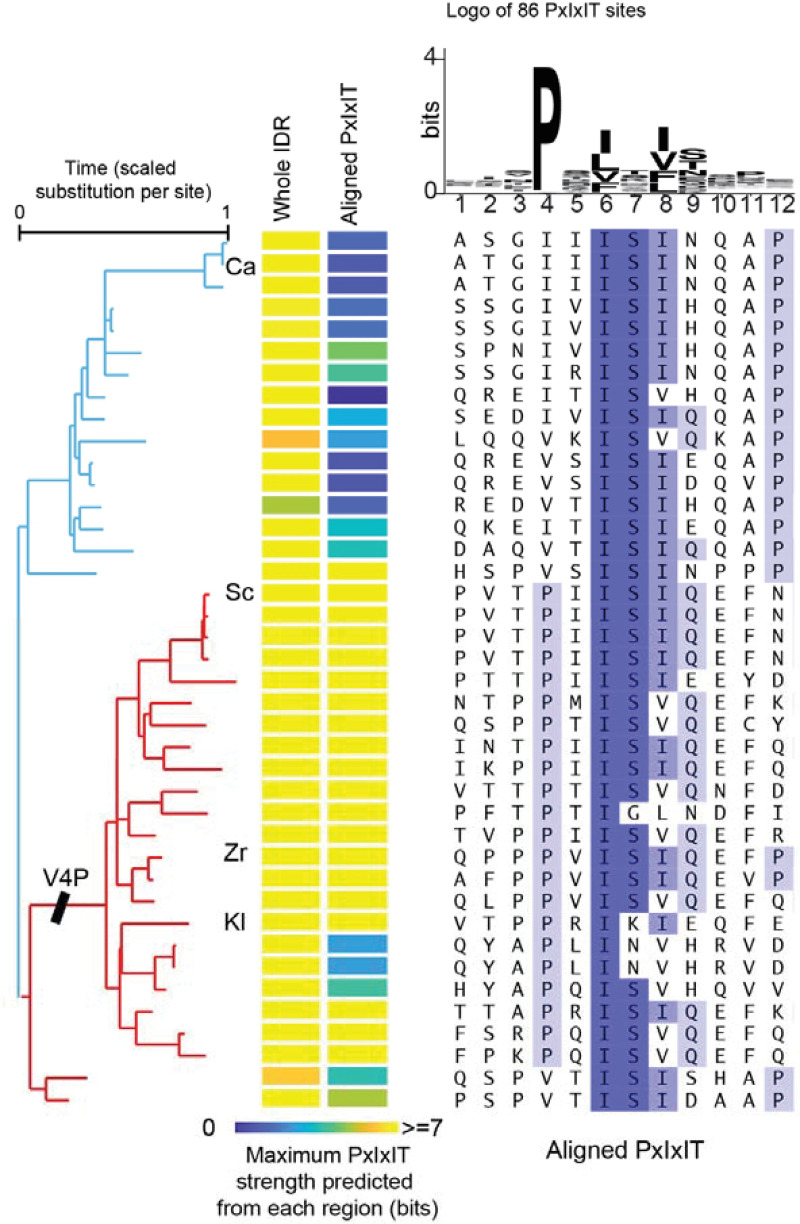
PxIxIT strength in the region homologous to the *S*. *cerevisiae* docking site is predicted to increase in the lineage leading to the Saccharomyces. The left heatmap represents the strength of the strongest PxIxIT site identified in the whole IDR. The right heatmap shows the predicted strength in the homologous region of the *S*. *cerevisiae* PxIxIT (shown in the alignment). The branch lengths of the phylogenetic tree are estimated by maximum likelihood based on alignments of the entire Crz1 protein. The label V4P indicates the branch where the inferred V4P substitution occurred. The alignment shows the 12 residues of 40 fungi. The logo represents the PSSM used.

We next sought to identify signaling interaction sites in the Crz1 IDRs that had changed during evolution. Previous research showed that increasing the affinity of one of the calcineurin docking sites, PxIxIT, leads to a higher pulsing frequency [25]. Therefore, we hypothesized that the PxIxIT strength of the Saccharomyces clade is higher than its sister clade that includes *C*. *albicans* and that this increased strength is needed for pulsing. To test this, we used a Position Specific Scoring Matrix (PSSM [55,56], see methods) to predict PxIxIT strength (S7A Fig, R^2^ = 0.74 between the measured affinity (K_d_) and the predicted PxIxIT strength of experimentally confirmed PxIxITs [56]) to predict the PxIxIT strength of Crz1 IDRs from 40 fungi of the Saccharomyces clade and the sister clade that contains *C*. *albicans*. Consistent with the conservation of calcineurin regulation of Crz1, most fungi contain at least one strong PxIxIT site somewhere in their IDRs (Maximum PxIxIT strength >7, Fig 4), indicating the PxIxIT strength alone cannot explain the functional difference between the IDRs from the Saccharomyces and *C*. *albicans*.

Previous research also showed a connection between the position of the calcineurin docking site and dephosphorylation rate [57]. When we aligned the 40 Crz1 IDRs [58], we found that three of the key residues in the *S*. *cerevisiae* PxIxIT site are mostly conserved in the alignment (IS[IV], Fig 4B) but the proline is not. The PxIxIT binding pocket of calcineurin buries the proline of the PxIxITs in hydrophobic residues [15,53,57], and as expected, the PSSM shows that proline is highly conserved in confirmed PxIxITs (Fig 4). We inferred a V-to-P substitution on the lineage leading to the Saccharomyces clade, which increases the predicted PxIxIT strength by ~4 bits (Fig 4), corresponding to a predicted reduction in K_d_ by ~400μM (S7A Fig). Therefore, we hypothesized that the increased strength of the *S*. *cerevisiae* PxIxIT leads to pulsing and the associated fitness benefit. Because rapidly evolving disordered regions are difficult to align, to rule out the possibility that species outside of the Saccharomyces actually do have a strong homologous PxIxIT site, we repeated this analysis using a 100-residue window around the *S*. *cerevisiae* docking site and found similar results: in this region of the IDR, only the Saccharomyces showed PxIxIT sites comparable in strength to *S*. *cerevisiae* (S7B Fig)

### Increasing PxIxIT strength in the homologous region of S. cerevisiae IDR is sufficient to rescue pulsing phenotype and fitness

**Fig 5:**
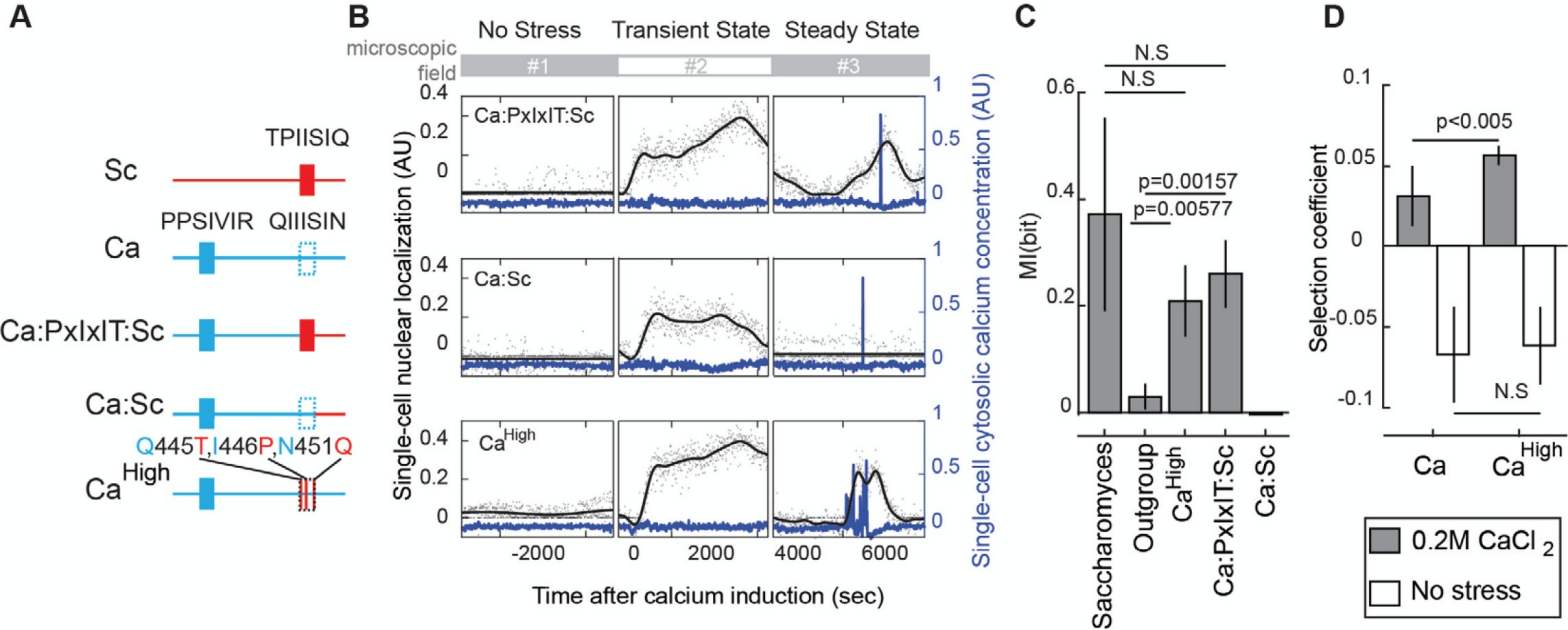
The *S*. *cerevisiae* PxIxIT strength in the specific part of IDR is sufficient for the *C*. *albicans* IDR to show a pulsing phenotype and improve fitness under 0.2M calcium stress. A) A schematic diagram of the PxIxIT sites on orthologous and chimeric IDRs. Sc: *S*. *cerevisiae* IDR; Ca: *C*. *albicans* IDR; Ca:PxIxIT:Sc: chimeric IDR of the N-terminal region of the *C*. *albicans* IDR (a.a. #1 to 444) and the C-terminal region of the *S*. *cerevisiae* IDR including PxIxIT site (a.a. #330 to 568); Ca:Sc: chimeric IDR of the N-terminal region of the *C*. *albicans* IDR (a.a. #1 to 451) and the C-terminal region of the *S*. *cerevisiae* IDR excluding PxIxIT site (a.a. #337 to 568); Ca^High^: *C*. *albicans* IDR with three point-mutations (Q445T, I446P, N451Q). B) Representative single-cell trajectories of cytosolic calcium concentration (blue lines) and nuclear localization (black lines), which are the Gaussian Process regression based on 600 time-points (black dots). Plots are broken to indicate when each trajectory composites a different cell from a different microscope field. C) Averaged mutual information between the calcium burst and the dynamics of pulsing reporters. P-values indicated are from a two-tail two-sample t-test. n = 7, 4, 3, 3, 1 experiments. Error bars represent 1.96 SE. D) The selection coefficient calculated from the competition assays under no stress or 0.2M calcium stress. n = 6 replicates for Ca and 16 replicates for Ca^High^ with three cell lines. Error bars represent 1.96 SE. P-values indicated are from a two-tail two-sample t-test. AU: Arbitrary Units.

We next tested if the increased PxIxIT strength in the homologous region of *S*. *cerevisiae* IDR is required for pulsing. We used time-lapse microscopy to investigate if the *S*. *cerevisiae* PxIxIT can introduce pulsatility into an outgroup IDR. First, we designed a chimeric IDR with the *S*. *cerevisiae* PxIxIT and C-terminal sequences but the N-terminal region of the *C*. *albicans* IDR (Fig 5A, denoted as Ca:PxIxIT:Sc). We note that, consistent with the analysis of PxIxIT strength above (Fig 4), the *C*. *albicans* IDR contains an additional strong PxIxIT in the N-terminal (PSIVIR, a.a.# 23~28), so this chimera actually contains two strong docking sites. Next, we made a chimera where we swapped the C-terminal portion of the *S*. *cerevisiae* IDR without the *S*. *cerevisiae* PxIxIT site. This chimera still retains the N-terminal *C*. *albicans* PxIxIT site (denoted as Ca:Sc, Fig 5A). Finally, we simply increased the predicted PxIxIT strength in the homologous region to the *S*. *cerevisiae* level via three point-mutations (Q445T, I446P, N451Q, denoted as Ca^High^, Fig 5A). This construct also contains two strong docking sites.

To determine if chimeric IDRs support pulsatility by responding to calcium bursts, we estimated the mutual information about the presence of calcium bursts from the dynamics of pulsing reporters (Fig 5B and 5C, more exemplary traces in S8 Fig). We found that the dynamics of both constructs with the *S*. *cerevisiae* docking site (Ca^High^ and Ca:PxIxIT:Sc) encoded more mutual information than that of the outgroup IDRs (mean MI = 0.21 bits and 0.26 bits, 2-tails t-test, p = 0.00577 and 0.00157, n = 3 and 3, respectively). In contrast, we did not find any evidence for the mutual information encoded in the dynamics of Ca:Sc (MI = -0.003 bits, n = 1). These results indicate that the *S*. *cerevisiae* PxIxIT strength is sufficient for the *C*. *albicans* IDR to support Crz1 pulsatility.

**Fig 6:**
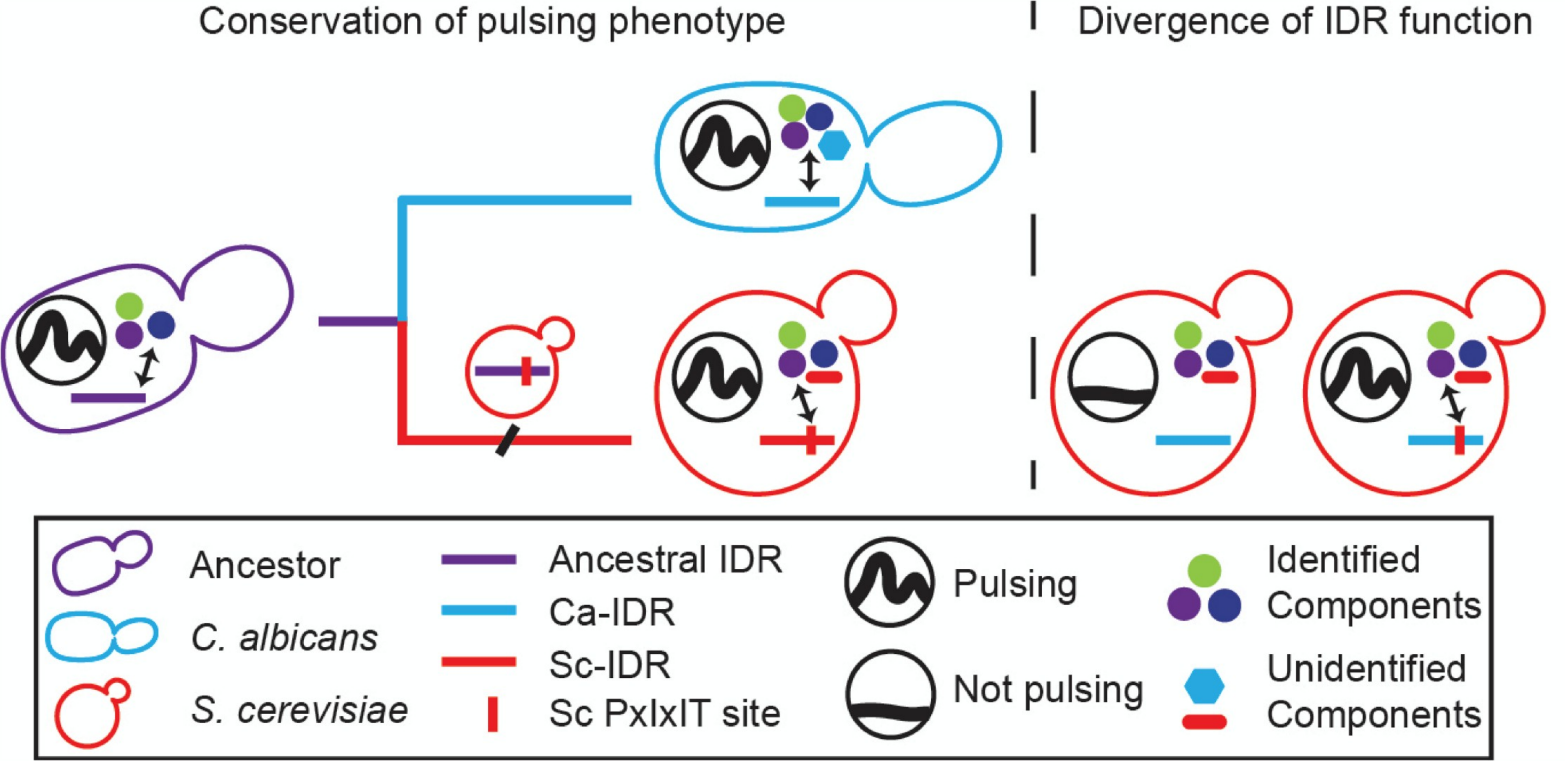
A schematic representation of the evolutionary model that Crz1 pulsing is conserved by stabilizing selection while the underlying mechanism of pulsing has been rewired. Identified components on the signaling pathway (i.e., calcineurin, karyopherin, represented by circles of different colors) are likely to interact with both Ca- and Sc-IDRs, and unidentified components (cyan and red shapes) are likely to interact specifically with each IDR.

Motivated by our finding that Saccharomyces IDRs rescue fitness in growth competition assays, we wondered whether the pulsatile dynamics of the Ca^High^-IDR improves fitness. Therefore, we performed the fitness assay with and without 0.2 M calcium stress and compared the relative fitness by Ca^High^- IDR and the *C*. *albicans* IDR. Previous research showed that Crz1 from *C*. *albicans* increased the growth rate of the *S*. *cerevisiae CRZ1* deletion strain [31]. Consistent with this, we found that cells expressing the *C*. *albicans* IDR showed a positive selection coefficient relative to *CRZ1* deletion strains (Fig 5D). Remarkably, the selection coefficient of Ca^High^-IDR expressing cells, relative to the same *CRZ1* deletion strains, was significantly higher than that of the cells expressing the *C*. *albicans* IDR (mean s = 0.057 vs. 0.031, 2-tails t-test, p = 0.0031, n = 16 and 6). In contrast, we did not find a significant difference between the selection coefficients in the absence of stress. These results support our hypothesis that the extra information encoded in the pulsatile dynamics improves fitness. Taken together, our experimental data suggest that the increase in the PxIxIT strength of *Saccharomyces* IDRs is one aspect of the signaling mechanisms that have changed, despite conservation of Crz1 pulsing (Fig 6).

## Discussion

Together, our results suggest that Crz1 pulsatility can be realized by highly divergent and functionally different IDRs. Our data are consistent with the idea that PxIxIT strength changes were one of the functionally compensatory changes in the calcium/Crz1 signaling pathway under stabilizing selection (Fig 6) [45]. Within the Saccharomyces, our results support the idea that highly diverged IDR sequences can support the same molecular function, consistent with the current model of stabilizing selection on molecular features preserving IDR function despite sequence divergence [43,44]. However, our results for more distantly related fungi go further: we found that for the distantly related fungi, the function of the IDRs is not preserved, even though the pulsing phenotype is preserved. This is consistent with an even broader view of stabilizing selection where compensatory changes between components of a conserved pathway occur to preserve a dynamic signaling phenotype. Furthermore, our observations illustrate how small evolutionary changes in IDRs can lead to (at least one) functional difference in the mechanism underlying stochastic signaling, ruling out the idea that the rapid sequence divergence is simply due to changes in non-functional residues. As one possible mechanism for compensatory change, we note that the regulatory domain of calcineurin subunit A (Cna1) is highly diverged (S9 Fig), intrinsically disordered, and interacts with calmodulin [59]. If these differences in the regulatory domain affect the probability of calcineurin activation by calmodulin, then Crz1 orthologues with a weak PxIxIT strength could still be dephosphorylated effectively and pulse. Under this scenario, the IDRs in distantly related fungi could exhibit pulsing dynamics that are not observed in our reporter system. More generally, our results are consistent with the idea that IDR sequences encode important functional information and are not “junk proteins” that evolve entirely randomly [44], but instead evolve under stabilizing selection [45,60] and accumulate rapid divergence at the sequence level due to the weak constraints relative to folded protein domains [42].

Our results also suggest Crz1 pulsatility transmits information beneficial for cell growth under 0.2M calcium stress. Although in principle, other effects on Crz1 function could lead to the fitness differences we observed when highly diverged IDRs are expressed in *S*. *cerevisiae*, the increase in both pulsing and fitness observed when we made three point mutations in the weak PxIxIT in the *C*. *albicans* Crz1 IDR suggests that pulsing is responsible for the fitness differences we observed. If Crz1 pulsing is indeed beneficial to cells under calcium stress, Crz1 pulsing is expected to be widely conserved, consistent with our observations of pulsatility in distantly related fungi. Although we cannot rule out a convergent evolution model, where Crz1 pulsing emerged independently in the lineages leading to *S*. *cerevisiae*, *C*. *albicans*, and *S*. *pombe*, to us, this is much less parsimonious than preservation of the phenotype by stabilizing selection.

Although the *C*. *albicans* IDR contains a PxIxIT consensus site in the N-terminal region, pulsing is only supported in *S*. *cerevisiae* when the PxIxIT strength of the homologous region to the *S*. *cerevisiae* PxIxIT (a.a.# 445 to 451) is increased, suggesting that calcineurin-dependent pulsatility depends on the position of PxIxIT. Effective dephosphorylation could be necessary for calcineurin-dependent pulsatility[19,23], and previous research has shown instances that the efficiency of dephosphorylation by calcineurin was affected by the distances between calcineurin docking sites and phosphorylated residues [57]. Hence, we suggest that evolution has fine-tuned signaling dynamics through the poorly understood position dependency of short linear motifs in this case.

Previous quantitative studies on dynamic signal processing focused on the information encoded by synchronous, transient dynamics [51,52,61]. Because all five IDRs in our study show transient nuclear localization after calcium induction (Fig 2A), we could measure the effect of steady state stochastic pulsing while minimizing the effects of differences in the transient dynamics. We not only show that the stochastic dynamics during steady state encode environmental information but also that information is transmitted between two stochastic components on the same pathway: the bursting dynamics of cytosolic calcium concentration and the pulsatile dynamics of Crz1 nuclear localization. Thus, we applied information theory to show information transmission between two unsynchronous and stochastic cellular dynamics.

## Methods

### Statistical analysis of disorder in pulsing transcription factors

We obtained a list of all transcription factors in *S*. *cerevisiae* from YeTFaSCo (de Boer, C.G., Hughes, T.R. (2011) and a list of all pulsing transcription factors in *S*. *cerevisiae* from a systematic microscopy screen (Dalal et al 2014). The proportion of residues in long intrinsically disordered regions in transcription factors was calculated based on DISOPRED3 [62] predictions after filtering out predicted sequences shorter than 30 amino acids. Transcription factors with no predicted IDRs were not included in the analysis.

### Yeast strains

Lasers used during fluorescence microscopy are known to induce nuclear localization of Crz1 and can affect the dynamics of the pulsing reporters [54]. To minimize these effects but still measure dynamics of both cytosolic calcium concentration and Crz1 nuclear localization, we designed double-reporter strains expressing the pulsing reporters described in the text (IDRs followed by yEGFP-tagged defective zinc finger, IDR-dZF-yEGFP) and the calcium reporter GCaMP3 [47]. Plasmids expressing the pulsing reporters were constructed using Gibson assembly protocol [63]. The pulsing reporter genes were assembled between the promoter of CRZ1 and the ADH1 terminator (pCRZ1-IDR-dZF-yEGFP-tADH1) and integrated at the *URA3* locus of reference strain BY4741 using a selectable marker (URA3). The fragments of orthologous IDRs were amplified from the genomic DNA of the corresponding species and corrected the CUG codon usage [64]. The IDR with the mSRR mutations was constructed by modifying the Sc-pulsing reporter plasmid (URA3::pCRZ1-ScIDR-dZF-yEGFP-URA3MX). The chimeric IDRs were constructed through two-fragment transformations, with each fragment amplified from the Sc-pulsing reporter plasmid (URA3::pCRZ1-ScIDR-dZF-yEGFP-URA3MX) or Ca-pulsing reporter plasmid (URA3::pCRZ1-CaIDR-dZF-yEGFP-URA3MX). In the same strains, we integrated a previously constructed pRPL39-GCaMP3-tADH1 at the *HO* locus using a selectable marker (LEU2) [19]. All transformations were performed using the standard lithium acetate procedure [65].

Compared to the previously constructed dual-color strains [19], the *S*. *cerevisiae* double-reporter strain required ~90% lower laser intensity (no observable nuclear localization induced by laser stress [54]) to record both the dynamics of cytosolic calcium concentration and Crz1 nuclear localization. Because of the spatial differences in the patterns (nuclear Crz1-GFP vs. cytoplasmic GCaMP3), the two signals can be distinguished using a two-component mixture model (described in the methods section of Reporter intensity quantification).

Each fitness assay strain was constructed by integrating an IDR and a wild type zinc finger at the endogenous locus of CRZ1 through two-fragment transformation using a selectable marker (HIS3). The zinc finger was tagged with yEGFP to report expression level. The IDR fragments were amplified from the existing plasmids expressing the corresponding pulsing reporters and zinc fingers or amplified from the genomic DNA of the corresponding double-reporter reporter strains. For the competition assay on plates (see below), each fitness assay strain was labeled with red by genomic integration of pRPL39-yemCherry-tADH1 at the promoter region of CAN1 using a selectable marker (LEU2).

The GFP-expressing *S*. *pombe* strain is a gift from Dr. Gordon Chua [30].

The *C*. *albicans* strain CaLC7415 with both copies of *CRZ1* C-terminally tagged with GFP was made using a transient CRISPR approach adapted from Min *et al*. [66]. The GFP-NAT cassette was PCR amplified from pLC389 using oLC9367 and oLC9368 (see tables below for plasmids and oligos). The CaCAS9 cassette was amplified from pLC963 using oLC6924 and oLC6925. The sgRNA fusion cassette was PCR amplified from pLC963 with oLC5978 (S1 Table for plasmids) and oLC9371 (fragment A) and oLC5980 and oLC9372 (fragment B) (S2 Table for pligos), and fusion PCR was performed on fragments A and B using the nested primers oLC5979 and oLC5981. The GFP-NAT cassette, sgRNA, and Cas9 DNA were transformed into SN95. Upstream integration was PCR tested using oLC600 and oLC9369, and downstream integration was tested using oLC274 and oLC9370. Lack of a wild-type allele was PCR tested using oLC9369 and oLC9373.

### Spinning-disk confocal microscopy and image analysis

A Nikon CSU-X1 was utilized for time-lapse imaging at room temperature (22°C) for *S*. *cerevisiae* and *S*. *pombe* strains. 488 nm laser was applied with time resolutions of 6 sec/frame, exposure time of 50 msec, and 25% laser intensity. Bright-field images with out-of-focus black cell edge were acquired every minute for cell segmentation and tracking. The growth conditions were based on a standard protocol [19,30]. A Zeiss Axio Observer was utilized for time-lapse imaging at room temperature (22°C) for *C*. *albicans* strain. 488 nm laser was applied with time resolutions of 30 sec/frame, exposure time of 100 msec, and 100% laser intensity. All the time-lapse imaging experiments were started when cells were in log-phase. Cells were cultured in YPD with a carbon source of 2% glucose overnight and immobilized via ConA in SC during time-lapse imaging. 35 mm glass bottom chambers and a stage chamber were applied for imaging and were covered with lids to prevent evaporation of the media and temperature fluctuation. Calcium stress was applied through a syringe. Time-lapse movies of every strain had been replicated on different days to control day-to-day variation and showed reproducible results.

Segmentation was automatically performed by YeastSpotter [67]. Cell tracking was performed by identifying 90% of overlapping cell areas between two frames. Mis-segmented and miss-tracked objects were manually removed. 100–300 cells were identified in each time-lapse movie. Single-cell photobleaching correction was conducted after single-cell reporter intensities were quantified (see below) using bi-exponential regression [68] with the baseline of the calcium reporter.

### Reporter intensity quantification

The cytosolic calcium concentration and transcription factor nuclear localization are defined as fold change relative to their basal levels (set as 0 arbitrary unit (AU)) and are quantified from single-cell time-lapse images. For each time point, The cytosolic intensity of the calcium reporter and the nuclear intensity of the pulsing reporter was quantified by fitting a mixture of a Gaussian distribution and a uniform distribution to the pixel intensities of each segmented cell area, and the parameters of distributions were estimated using expectation-maximization (see the supplementary text of [19] for more details and derivation of the algorithm). Once the parameters were estimated, the estimate of the calcium reporter at a time point is the mean of the Gaussian distribution, and the estimate of nuclear localization is the difference between the means of the uniform distribution and the Gaussian distribution. The algorithm can reproduce previous observations from time-lapse movies where Crz1-RFP and GCaMP3 are merged into one channel S10 Fig.).

### Competition fitness assay on plates

Previous research showed that competition assays could be robustly performed through plate readers [69], which provides both the sensitivity of competition assays [43,70] and the high performance of growth assay on plates [71]. We followed this approach and recorded competition of fitness strains with a plate reader. Cells were grown in SC media at 30°C for ~48 hours and then serially diluted to 1/1024 of the initial concentration on a flat-bottom 96-well plate. The plate reader Tecan M1000 was automatically run by the application Tecan i-Control. OD600 and RFP intensity were measured every 15 minutes for 24 hours at 30°C, and the plates were constantly shaken through the whole experiment. Similar to the protocol of competition-based fitness assay on plates [69], the wells on the plates were either monoculture or mixed-culture. The mono-cultural wells contained only the RFP-labeled strain and were for the calibration between RFP intensity and OD600 absorbance through linear regression. The mixed-cultural wells contained both the RFP-labeled strain and the colorless strain. The absorbance of the colorless strains in the mixed-culture wells was estimated by subtracting the measured absorbance by the absorbance inferred from the RFP intensity, so the growth curves of both competing strains in each mixed-culture well can be obtained. The time point when the growth curves reached the 10^th^ generation was identified and kept consistent throughout each experiment. The relative selection coefficients were calculated with the formula [72,73]

$$\frac{\ln\frac{EXP_{t}}{WT_{t}}-\ln\frac{EXP_{t}}{WT_{t}}}{t}=\ln\;(1+s),$$

where *t* means the number of generations and *s* is the selection coefficient.

We found that this approach provides a resolution of the selection coefficient to 10^−3^ and successfully reproduced a previously reported small fitness defect (S11 Fig).

### Single-cell trajectory quantification with Gaussian Process

In the previous studies of pulsatile transcription factors, pulses were identified before quantification and statistical analysis, e.g., pulse frequency[20,25] and pulse triggered averaging [50]. This approach presumes that the dynamics are pulsatile. However, in our case, whether a pulsing reporter pulse or not was to be determined. Therefore, we needed a more general approach to quantify single-cell trajectories.

We used a Gaussian Process regression model [19,49,74] with the squared exponential kernel to summarize each single-cell trajectory. The kernel can be expressed as

$$k(x_{1},x_{2})=a^{2}\exp\;(-\frac{{\left(x_{1}-x_{2}\right)}^{2}}{2l^{2}})$$

where *x*_1_, *x*_2_ indicate a pair of nuclear localization scores at different time points, *a* determines the average distance of the trajectory away from its mean, and *l* determines the length of the fluctuation on the trajectory. We used the default MATLAB (Mathworks) function for the Gaussian process, fitrgp. Estimation was considered numerically unstable if ln(*a*)<−6, and cell trajectories were removed if their estimates were below this value.

### Estimation of mutual information and pulse-triggered averaging

Information theory provides a natural framework[75] to quantify information transmission in cells as mutual information (MI). Previous studies estimated MI encoded in signaling pathways [51,52] with decoding methodology (e.g., an SVM classifier [52]). Here we adopted a widely used kNN estimator (k = 4, [51,76]) for the advantage of its simplicity. Two different parametric forms of trajectories (described below) were applied to estimate the MI between the calcium stress and the dynamics of pulsing reporters and the MI between the calcium bursts and the dynamics of pulsing reporters.

To estimate MI between the calcium stress and the dynamics of pulsing reporters, single-cell trajectories of pulsing reporters from one experimental replicate were parameterized with the Gaussian process regression model described above. Gaussian Processes normalizes the trajectories to their mean amplitudes, excluding information about the absolute level of nuclear localization. For each environmental condition (no stress or 0.2 M calcium stress), an equal number of trajectories were randomly selected and parameterized. The selected data were processed into *D* = {(**x**_1_, *y*_1_), (**x**_2_, *y*_2_),…,(**x**_*n*_, *y*_*n*_)} that consist of *i* = 1,…,*n* pairs of parameter values, x_*i*_ = {*a*_*i*_, *l*_*i*_}, and their corresponding environmental labels of 2 discrete values, *y*_*i*_*ϵ*{*c*_0_, *c*_1_} (no stress or 0.2 M calcium stress). **x** was jittered to avoid identical samples. The estimated MI (**x**; *y*) of one experimental replicate was bootstrapping 60 times for the average value.

To estimate MI between calcium bursts and the dynamics of pulsing reporters, we first categorize time points on each calcium trajectory into two groups. A typical trajectory of calcium reporter contains two types of fluctuations: a baseline of slow fluctuation and calcium bursts as rapid and large fluctuation. Our previous research showed that only calcium bursts lead to Crz1 pulses [19]; hence, the information about the presence of calcium bursts should be encoded in a short period of the pulsing dynamics after the bursts. Precisely, let a trajectory of pulsing reporter ***x*** = {*x*_1_, *x*_2_,…,*x*_*p*_} consist of *t* = 1,…,*p* nuclear localization scores, a block of the trajectory *X* = {*x*_*t*+1_, *x*_*t*+2_,…,*x*_*t*+*τ*_} should encode the information about the states of preceding calcium trajectory at time *t* (calcium burst or basal fluctuation) with the mean score 〈*X*〉 and the mean velocity of the score $\langle \dot{X}\rangle$. Our goal is to process data extract from j = 1,…,*m* blocks (*X*_*j*_ = {*x*_*t*+1+*τ*(*j*−1)_, *x*_*t*+2+*τ*(*j*−1)_,…,*x*_*t*+*τj*_}) such that *D*_*j*_ = {(**x**_1*j*_, *y*′_1_), (**x**_2*j*_, *y*′_2_),…,(**x**_*nj*_, *y*′_*n*_)} consist of *i* = 1,…,*n* pairs of statistic summaries, $x_{ij}=\{{\langle X_{j}\rangle }_{i},{\langle {\dot{X}}_{j}\rangle }_{i}\}$, and their corresponding burst labels of 2 discrete values, *y*′_*i*_*ϵ*{*c*′_0_*c*′_1_} (no calcium burst or calcium burst). We then estimate MI_*j*_(**x**_*j*_, *y*′) as the information encoded in the *j*th block of pulsing trajectory after a calcium fluctuation. The details of the pipeline are provided in S1 Text. We found that the estimation from the first block, MI_1_, is representative of each experimental replicate and reported MI_1_ in the results section.

We use a similar pipeline to adapt pulse-triggered averaging by simply averaging all the trajectories of pulsing reporters in a 20-min window centered around every labeled calcium burst.

### Sequence analyses

To predict the calcineurin docking strength of a sequence, we used a Position Specific Scoring Matrix (PSSM). PSSM is a standard statistical model widely used for predicting transcription factor binding strength of a DNA motif [77] or protein binding strength of a short linear motif in an IDR [55,56,78]. In this study, a PSSM was constructed with 86 experimentally confirmed calcineurin binding sites (so-called PxIxIT sites) collected from the ELM database [79] and other sources [27,46,56,80]. The calcineurin docking site of Crz1 was excluded to avoid circularity. The docking strength *S* of a sequence *X* with sequence length *w* was calculated as

$$S=\overset{w}{\underset{i=1}{\sum }}\underset{b}{\sum }X_{ib}\log\;(\frac{f_{ib}}{g_{b}}),$$

Where *b*∈**A**. **A** indicates the 20 amino acids, *X*_*ib*_ = 1 if the sequence is amino acid *b* at position *i* and 0 otherwise, *f*_*ib*_ is the probability of observing amino acid *b* at position *i* in a calcineurin docking site (from the PSSM), and *g*_*b*_ is the probability of observing amino acid *b* in the genomic background distribution and was assumed to 1/20. To test how well this simple model predicts measured calcineurin binding affinity, we compared the predicted strength, *S*, to the affinity of 10 characterized PxIxIT sites [56] and found that a linear model where a change of 1 unit of *S* (which is measured in bits) corresponds to 74.4 unit of K_d_ (measured in μM, SE = 15.4, p = 0.001).

## Supporting information

S1 Fig The proportion of predicted intrinsic disorder in pulsing transcription factors is significantly more than that of non-pulsing transcription factors. n = 216 vs 9 (non-pulsing vs pulsing). Dashed line represents the 0.6 threshold that we used to define “highly disordered”. According to this, 6 out of 9 pulsing transcription factors and 41 out of 216 non-pulsing transcription factors are highly disordered.
(PDF)

S2 Fig The IDR of Crz1 is sufficient for pulsing. A) Representative images of GFP channel and bright-field channel of three strains expressing endogenous Crz1 tagged with GFP (Crz1-GFP), a passive reporter of the IDR tagged with GFP (zinc fingered removed, ΔZF-reporter), and a passive reporter of *S*. *cerevisiae* Crz1 with defective zinc fingers (Sc-reporter), respectively. B) Representative trajectories of each strain. The trajectories of nuclear localization (black lines) are the Gaussian process regression based on nuclear localization score of 600 time-points (cyan dots).
(PDF)

S3 Fig Example traces single-cell trajectories of cytosolic calcium concentration (blue lines) and steady state nuclear localization in the passive reporter system (color-coded lines), estimated by Gaussian Process regression on 600 time-points (color-coded dots). (PDF)

S4 Fig Mutations in the conserved phosphorylation sites in the serine-rich region led to constant nuclear localization after 0.2M calcium induction and fitness defect under 0.2M calcium stress. A) Upper panels show the population average of nuclear localization score. Shadow indicates SD with n > 100 for each strain. Lower panels show representative single-cell trajectories of cytosolic calcium concentration (blue lines) and nuclear localization (black lines), which are the Gaussian Process regression based on 600 time-points (red dots). Plots are broken to indicate when each experiment switched to a different microscope field to avoid laser-induced nuclear localization. B) The selection coefficient obtained from the competition assays under 0.2M calcium stress (grey bars) or no stress (white bars). Error bars represent 1.96 SE. n > 10 replicates for each competition assay with at least three cell lines.
(PDF)

S5 Fig Example growth curves from a competition assay. Reference strain (Sc) and the strain expressing Ca-IDR (Ca) were cultured in 24 wells on a 96-well plate for 24 hours, where 12 wells contain normal media (no stress) and 12 wells contain media of 0.2 calcium stress. In this competition assay, Sc was tagged with RFP for estimating OD of the reference strain, and the OD of Ca was calculated by subtracting the total OD by OD of the reference strain. Lines represent each strain’s OD for the first 50000 sec from each well. Box represents the time period when time-lapse microscopy was performed (3 hours of inoculation from overnight culture followed by 3 hours of microscopy).
(PDF)

S6 Fig Example traces of CaCrz1 (A) and Prz1 (B) dynamics in the native systems during steady state before and after the addition of 0.2 M extracellular calcium. Dynamics of Prz1 are recorded from two nuclei of the same cell (orange and yellow lines) or form the only nucleus of a cell (brown line).
(PDF)

S7 Fig Calculate PxIxIT strength with PSSM. A) Predicted PxIxIT strength plotted against the measured PxIxIT affinity of the same sequences from the database of ref [56]. Linear regression model: y ~ 779–74.4x, R^2^ = 0.74. B) Heatmaps represent the maximum PxIxIT strength calculated from the sub-sequences of the whole IDR or the 100-residue homologous region around the *S*. *cerevisiae* PxIxIT.
(PDF)

S8 Fig Example traces of cytosolic calcium concentration (blue lines) and nuclear localization of chimeric IDRs (black lines), which are the Gaussian Process regression based on 600 time-points (black dots). (PDF)

S9 Fig The regulatory domain is one of the diverged regions on calcineurin. Calcineurin contains two subunits, calcineurin A (Cna1, A) and calcineurin B (Cnb1, B). Boxes on Cna1 alignment label the homologous regions of known domains.
(PDF)

S10 Fig Two dynamics recorded with two-color images and merged into one-color images can be distinguished via a mixture model. A) An example of single-cell trajectories before (upper plot) and after (lower plot) merging for Crz1 (dots) and calcium (blue trace). Black traces are Crz1 trajectories smoothed with Sacitzky-Golay filtering. Black circles indicate Crz1 pulses. B) Calcium bursts (left plot) and Crz1 pulses (right plot) identified in the experiments sorted from large to small. C) The distributions of the change in the number of identified Crz1 pulses after merging. D) The probability of first, second, third, and fourth Crz1 pulses plotted as a function of the time they occur relative to calcium bursts from the same cells. The left and the right stacked histograms are data from the separate images and the merged images, respectively. E) Data are divided into three groups based on calcium burst sizes and aligned to each group’s mean calcium burst size. The dots’ size represents the probability of finding a number of Crz1 pulses in a group (summed up to 1 in each column).
(PDF)

S11 Fig The competition fitness assay on 96-well plates can reproduce the significant growth defect of a mutant (noted as ‘5A’) reported by Zarin et al., 2017. A) Fluorescent intensity of the monoculture. Each marker represents the fluorescent intensity and OD of a well at each time-point. B) Mean selection coefficient calculated from the fluorescent data of mixed culture. Error bars represent 1.96 SE. Dashed line indicates the selection coefficient of 5A mutant reported by Zarin et al., 2017 (-0.038) C) The growth curves of WT and 5A strains in the mixed cultures predicted by the algorithm of Ram et al., 2019. The selection coefficient is -0.05, which is calculated with the first and the last time point of the predicted growth curves.
(PDF)

S1 Text Detailed pipeline of MI estimation and species included for PxIxIT strength calculation. (DOCX)

S1 Table Plasmids used for constructing CaCrz1-GFP strain. (DOCX)

S2 Table Oligos used for constructing CaCrz1-GFP strain. (DOCX)

S1 Movie An example of CaCrz1 pulsing in *C*. *albicans*. (GIF)

S2 Movie An example of Prz1 pulsing in *S*. *pombe*. (GIF)

S1 Data Numarical data underlying the main figures. (XLSX)
