# Supplementary figures and images for: A functionally divergent intrinsically disordered region underlying the conservation of stochastic signaling
Source: PLoS Genet. 2021 Sep 10;17(9):e1009629. doi: 10.1371/journal.pgen.1009629 (PMC8457507; doi:10.1371/journal.pgen.1009629)

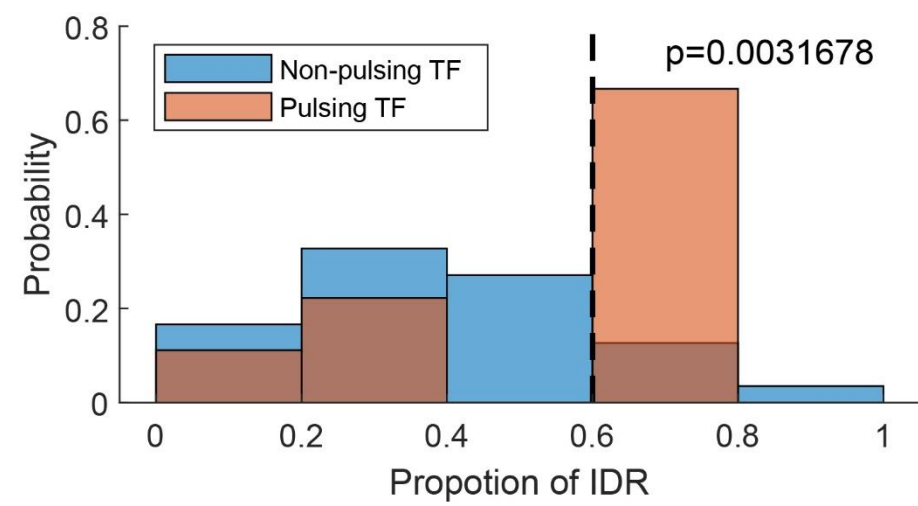

Supplement: S1 Fig — n = 216 vs 9 (non-pulsing vs pulsing). Dashed line represents the 0.6 threshold that we used to define “highly disordered”. According to this, 6 out of 9 pulsing transcription factors and 41 out of 216 non-pulsing transcription factors are highly disordered. (PDF) [file pgen.1009629.s001.pdf]

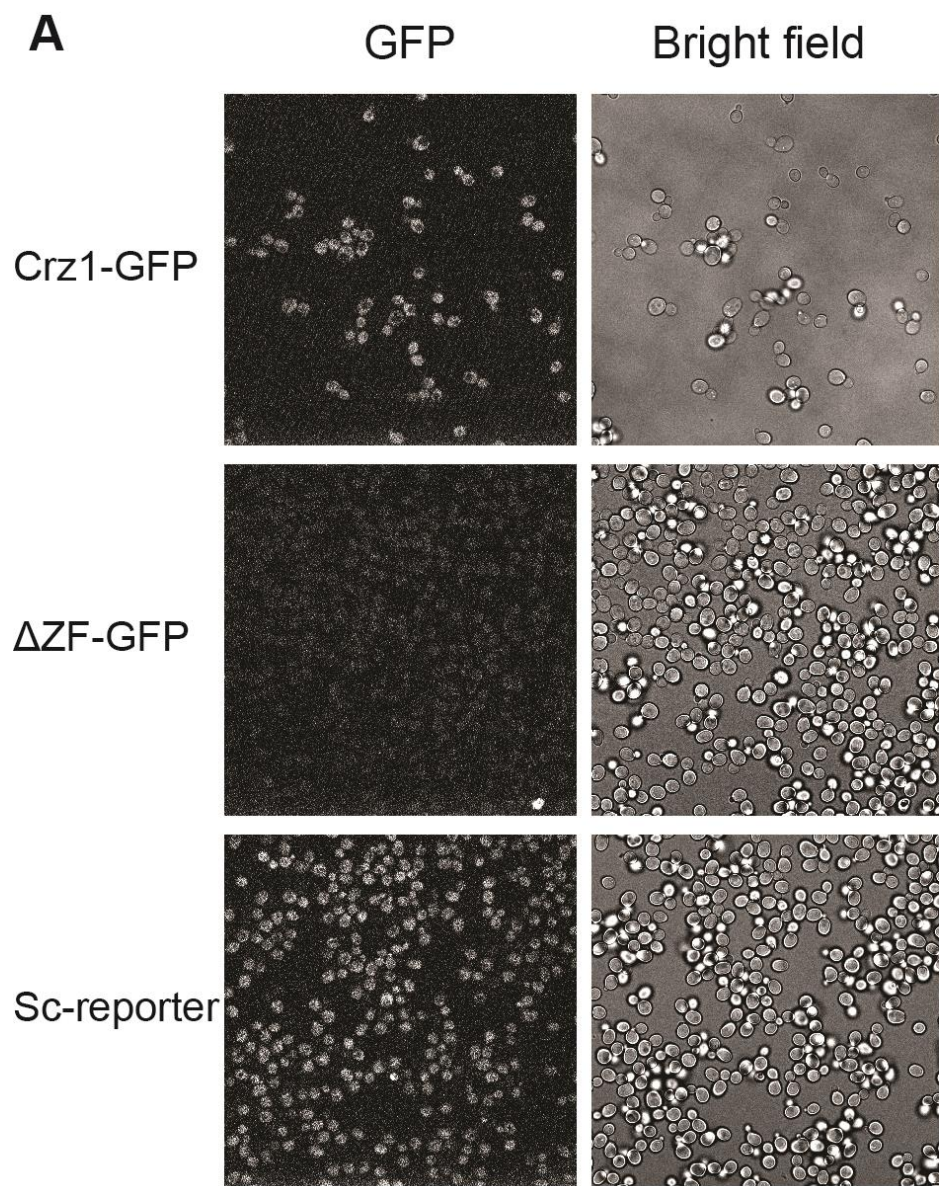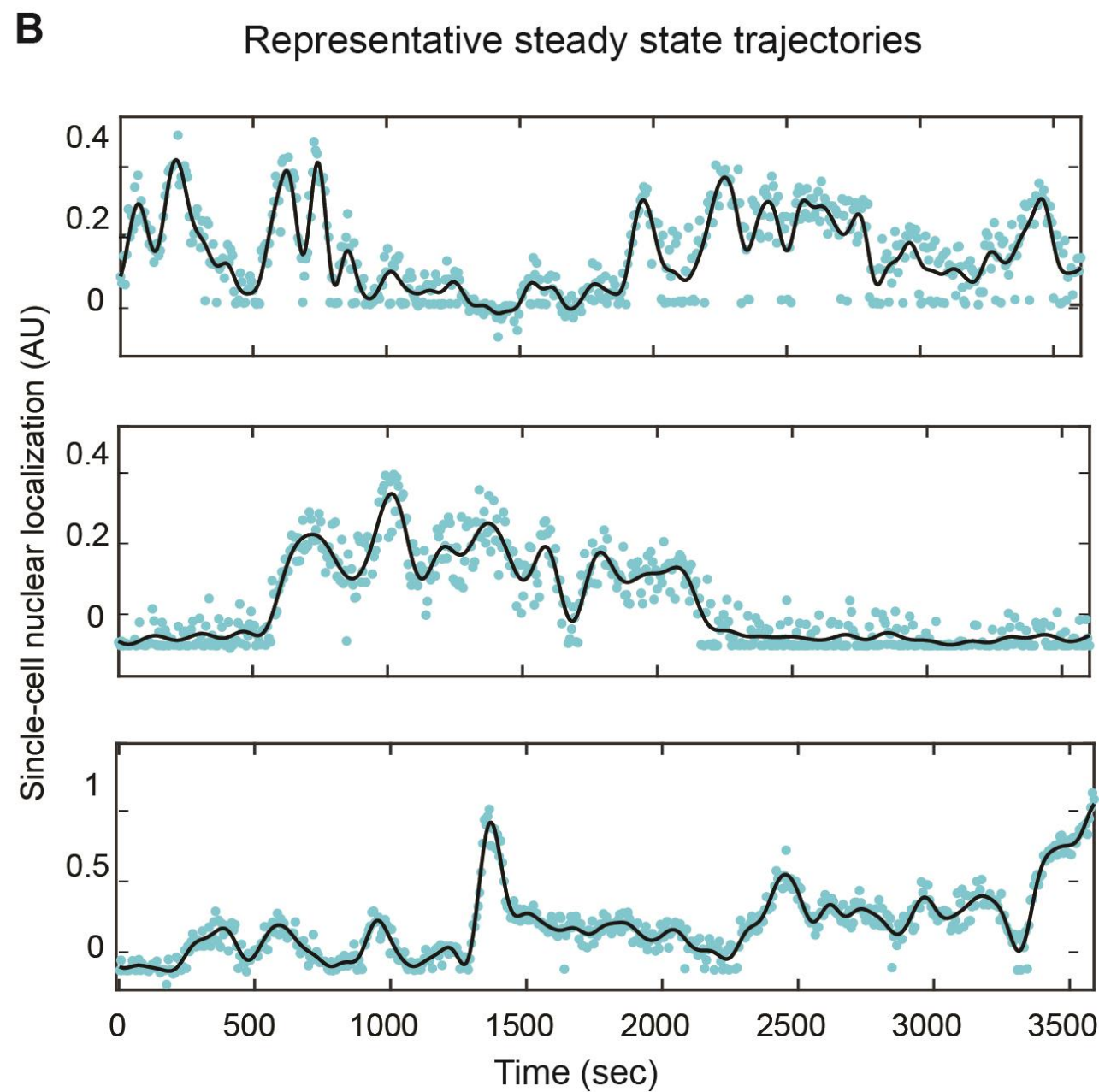

Supplement: S2 Fig — A) Representative images of GFP channel and bright-field channel of three strains expressing endogenous Crz1 tagged with GFP (Crz1-GFP), a passive reporter of the IDR tagged with GFP (zinc fingered removed, ΔZF-reporter), and a passive reporter of S. cerevisiae Crz1 with defective zinc fingers (Sc-reporter), respectively. B) Representative trajectories of each strain. The trajectories of nuclear localization (black lines) are the Gaussian process regression based on nuclear localization score of 600 time-points (cyan dots). (PDF) [file pgen.1009629.s002.pdf]

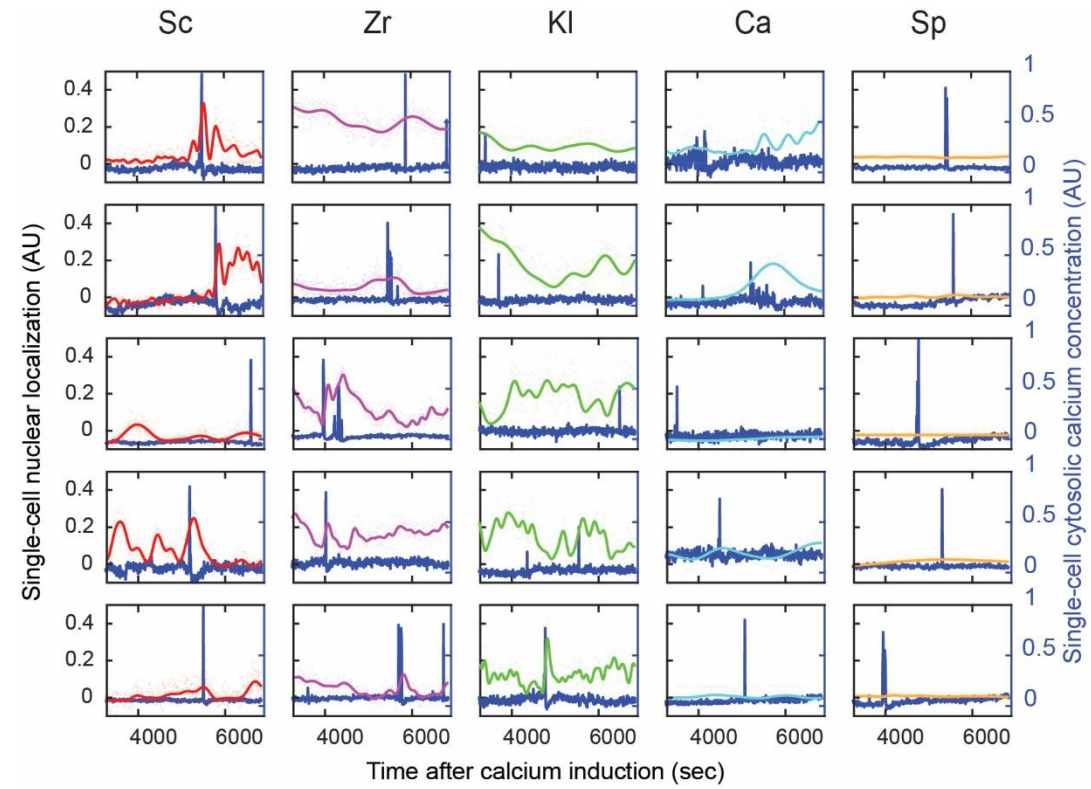

Supplement: S3 Fig — (PDF) [file pgen.1009629.s003.pdf]

**A**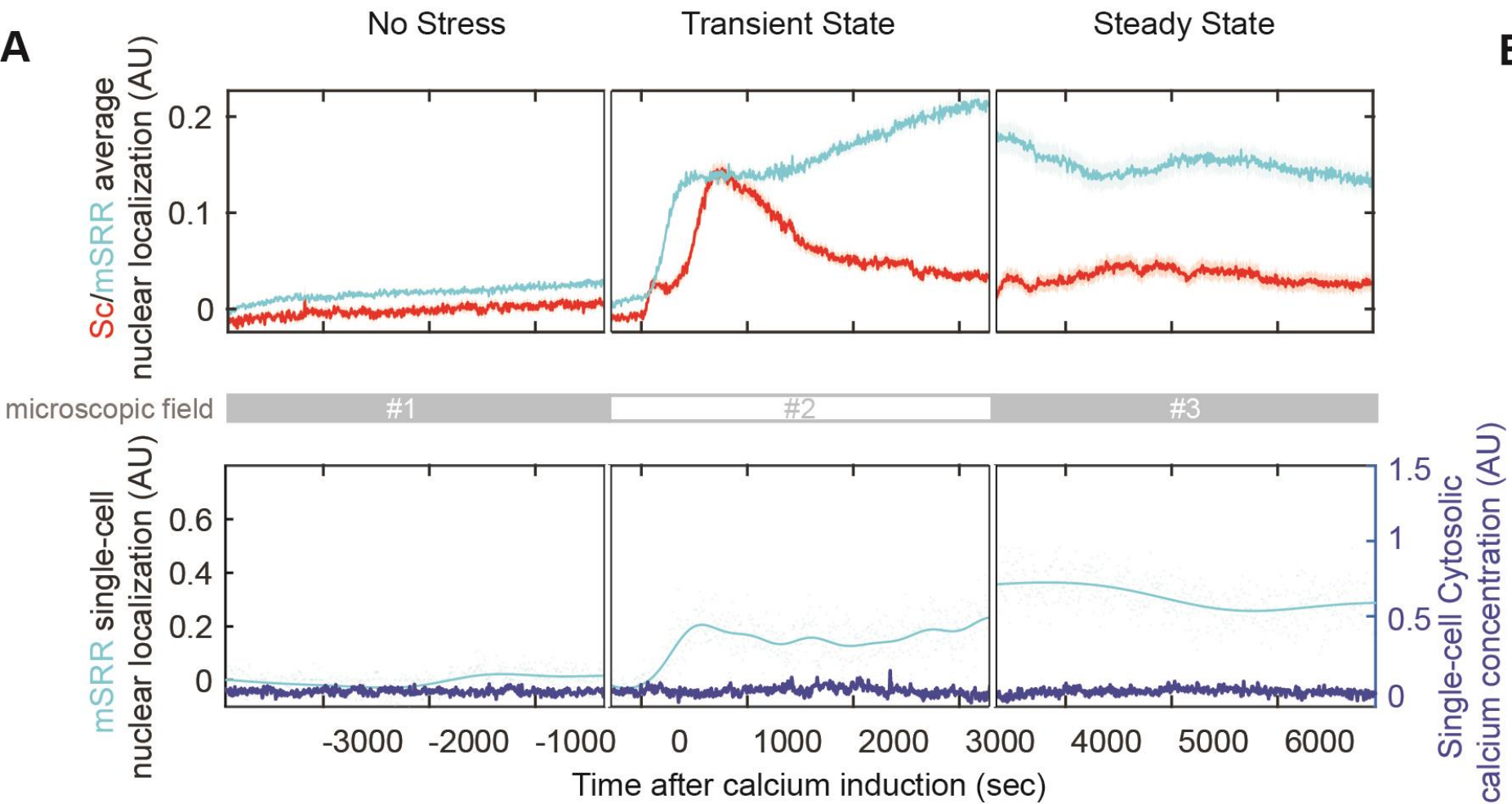**B**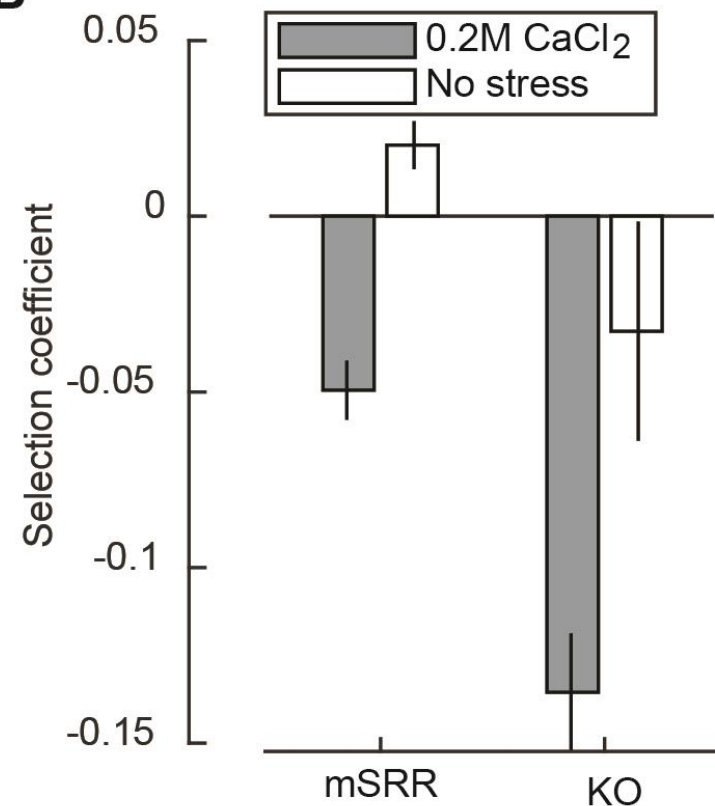

Supplement: S4 Fig — A) Upper panels show the population average of nuclear localization score. Shadow indicates SD with n > 100 for each strain. Lower panels show representative single-cell trajectories of cytosolic calcium concentration (blue lines) and nuclear localization (black lines), which are the Gaussian Process regression based on 600 time-points (red dots). Plots are broken to indicate when each experiment switched to a different microscope field to avoid laser-induced nuclear localization. B) The selection coefficient obtained from the competition assays under 0.2M calcium stress (grey bars) or no stress (white bars). Error bars represent 1.96 SE. n > 10 replicates for each competition assay with at least three cell lines. (PDF) [file pgen.1009629.s004.pdf]

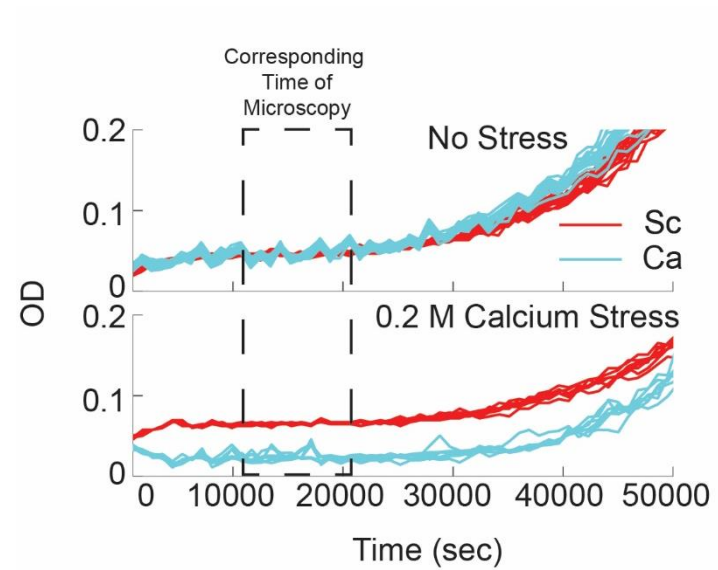

Supplement: S5 Fig — Reference strain (Sc) and the strain expressing Ca-IDR (Ca) were cultured in 24 wells on a 96-well plate for 24 hours, where 12 wells contain normal media (no stress) and 12 wells contain media of 0.2 calcium stress. In this competition assay, Sc was tagged with RFP for estimating OD of the reference strain, and the OD of Ca was calculated by subtracting the total OD by OD of the reference strain. Lines represent each strain’s OD for the first 50000 sec from each well. Box represents the time period when time-lapse microscopy was performed (3 hours of inoculation from overnight culture followed by 3 hours of microscopy). (PDF) [file pgen.1009629.s005.pdf]

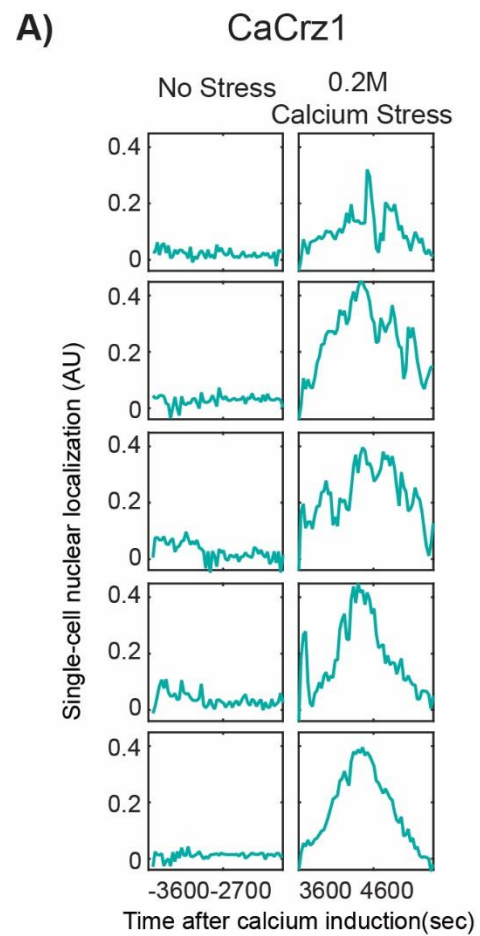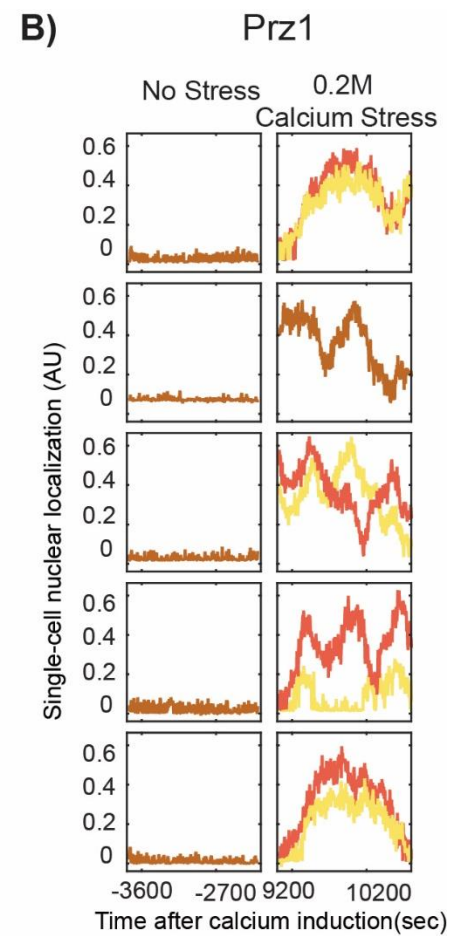

Supplement: S6 Fig — Example traces of CaCrz1 (A) and Prz1 (B) dynamics in the native systems during steady state before and after the addition of 0.2 M extracellular calcium. Dynamics of Prz1 are recorded from two nuclei of the same cell (orange and yellow lines) or form the only nucleus of a cell (brown line). (PDF) [file pgen.1009629.s006.pdf]

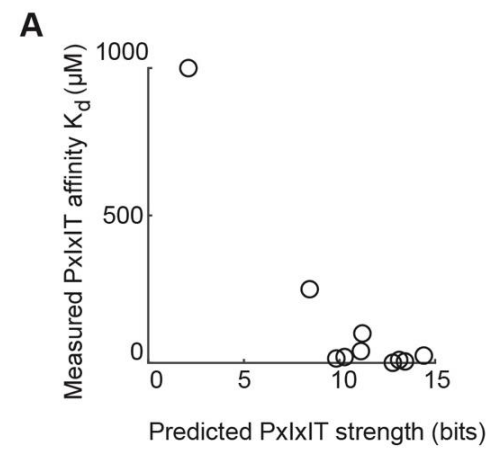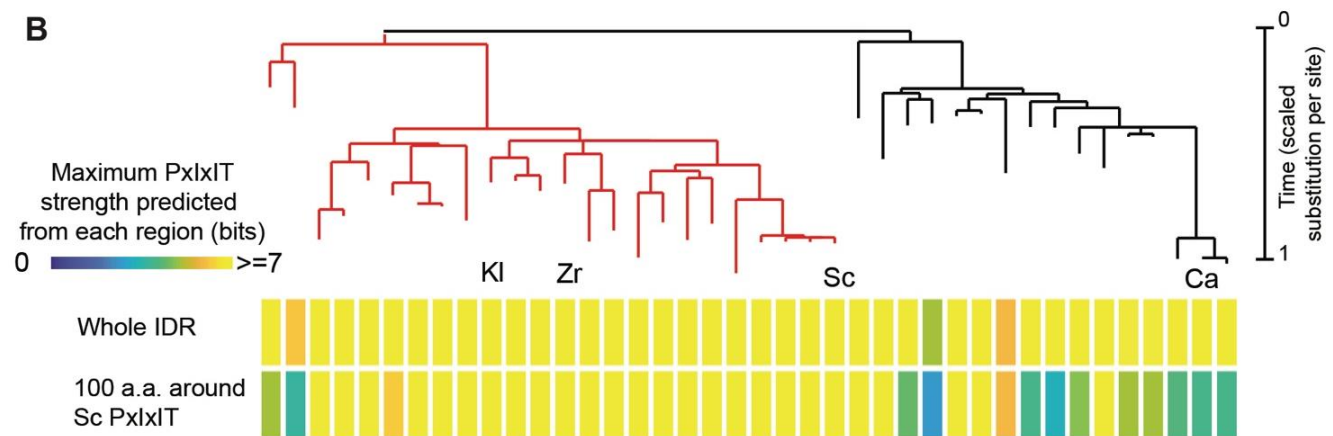

Supplement: S7 Fig — A) Predicted PxIxIT strength plotted against the measured PxIxIT affinity of the same sequences from the database of ref [56]. Linear regression model: y ~ 779–74.4x, R2 = 0.74. B) Heatmaps represent the maximum PxIxIT strength calculated from the sub-sequences of the whole IDR or the 100-residue homologous region around the S. cerevisiae PxIxIT. (PDF) [file pgen.1009629.s007.pdf]

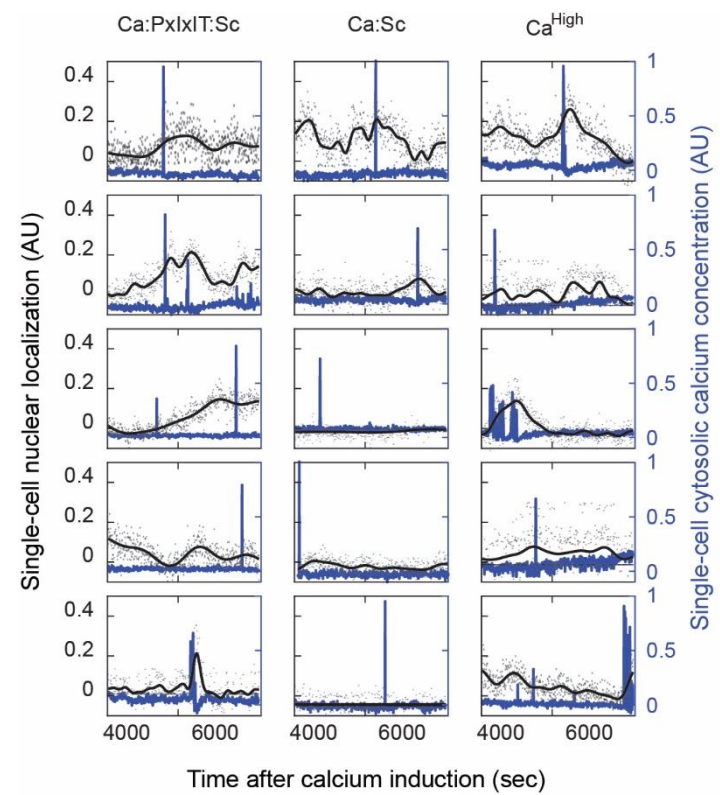

Supplement: S8 Fig — (PDF) [file pgen.1009629.s008.pdf]

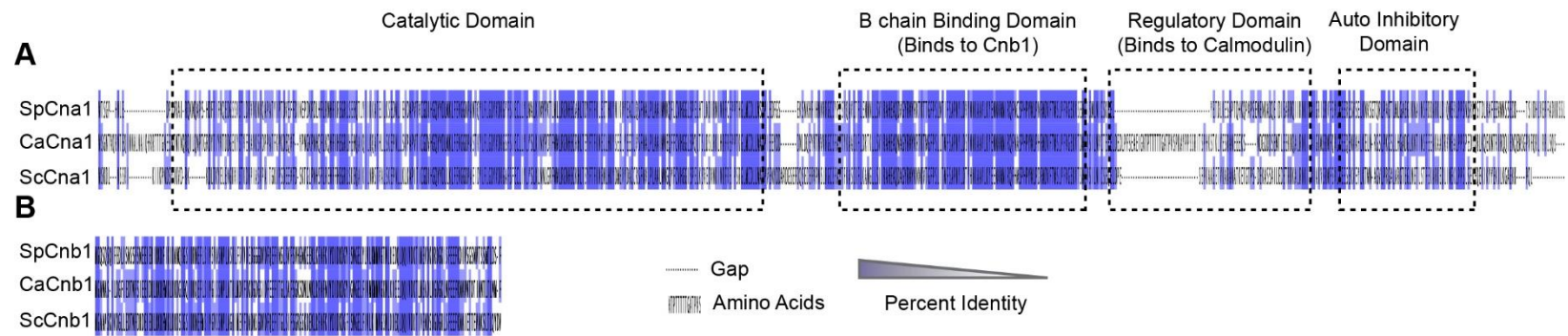

Supplement: S9 Fig — Calcineurin contains two subunits, calcineurin A (Cna1, A) and calcineurin B (Cnb1, B). Boxes on Cna1 alignment label the homologous regions of known domains. (PDF) [file pgen.1009629.s009.pdf]

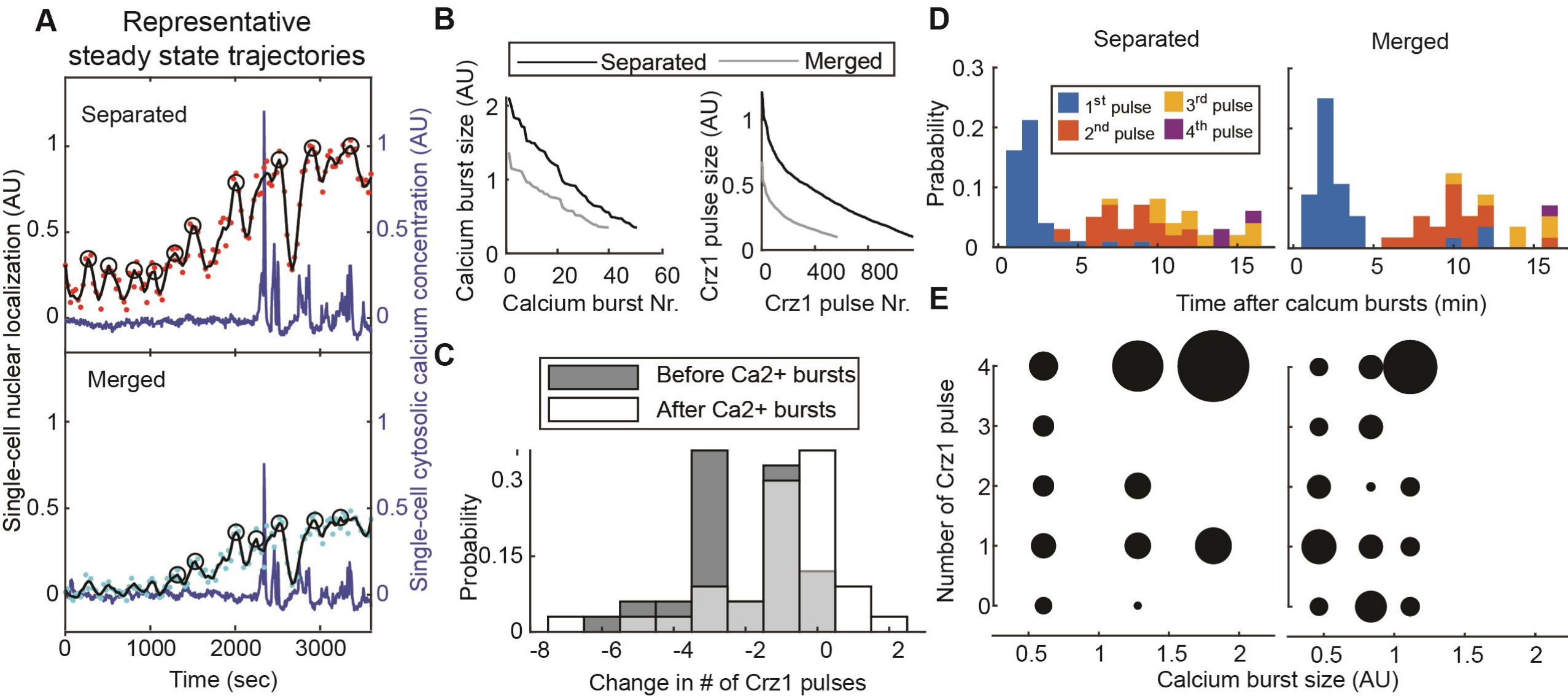

Supplement: S10 Fig — A) An example of single-cell trajectories before (upper plot) and after (lower plot) merging for Crz1 (dots) and calcium (blue trace). Black traces are Crz1 trajectories smoothed with Sacitzky-Golay filtering. Black circles indicate Crz1 pulses. B) Calcium bursts (left plot) and Crz1 pulses (right plot) identified in the experiments sorted from large to small. C) The distributions of the change in the number of identified Crz1 pulses after merging. D) The probability of first, second, third, and fourth Crz1 pulses plotted as a function of the time they occur relative to calcium bursts from the same cells. The left and the right stacked histograms are data from the separate images and the merged images, respectively. E) Data are divided into three groups based on calcium burst sizes and aligned to each group’s mean calcium burst size. The dots’ size represents the probability of finding a number of Crz1 pulses in a group (summed up to 1 in each column). (PDF) [file pgen.1009629.s010.pdf]

**A**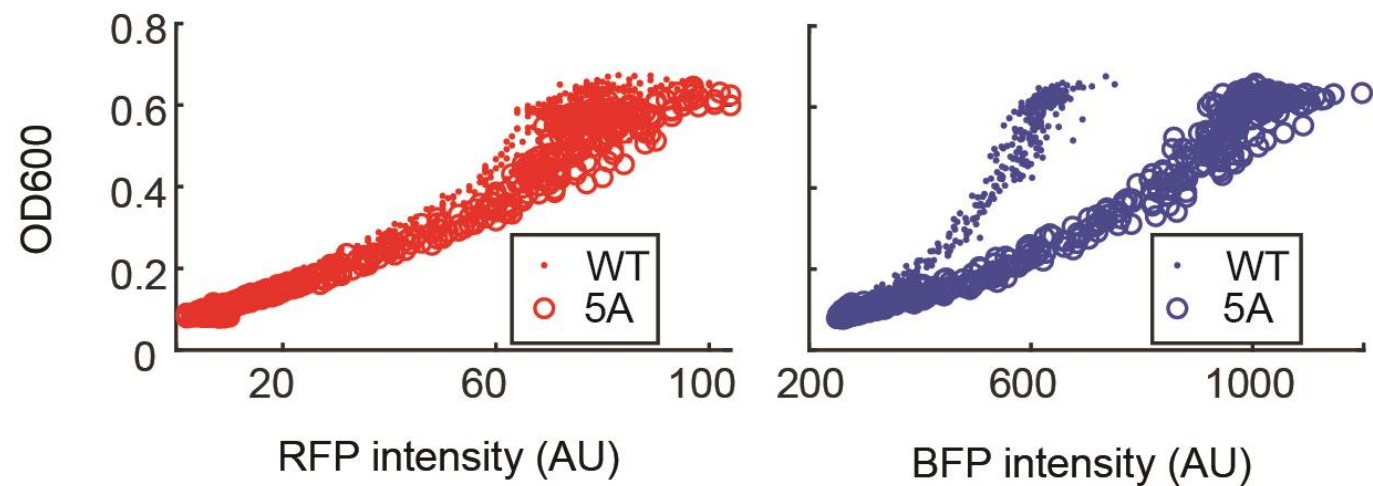**B**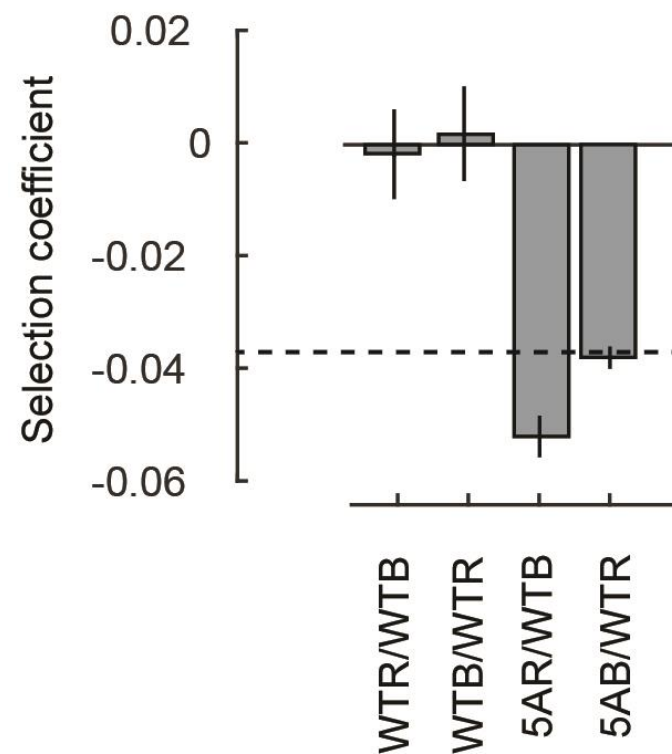**C**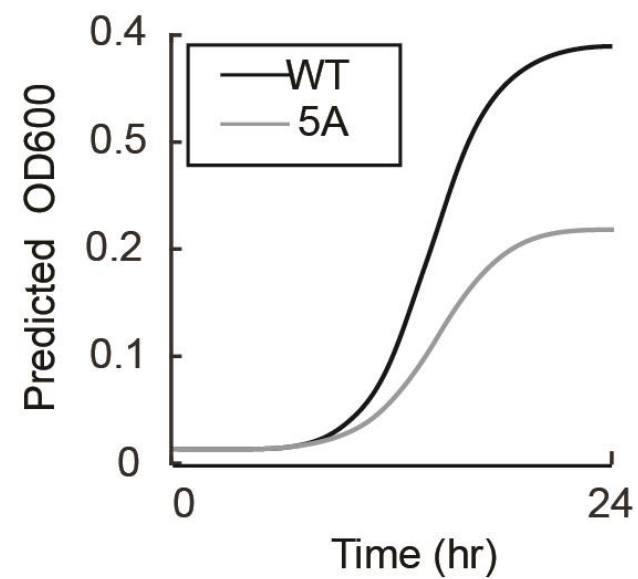

Supplement: S11 Fig — A) Fluorescent intensity of the monoculture. Each marker represents the fluorescent intensity and OD of a well at each time-point. B) Mean selection coefficient calculated from the fluorescent data of mixed culture. Error bars represent 1.96 SE. Dashed line indicates the selection coefficient of 5A mutant reported by Zarin et al., 2017 (-0.038) C) The growth curves of WT and 5A strains in the mixed cultures predicted by the algorithm of Ram et al., 2019. The selection coefficient is -0.05, which is calculated with the first and the last time point of the predicted growth curves. (PDF) [file pgen.1009629.s011.pdf]

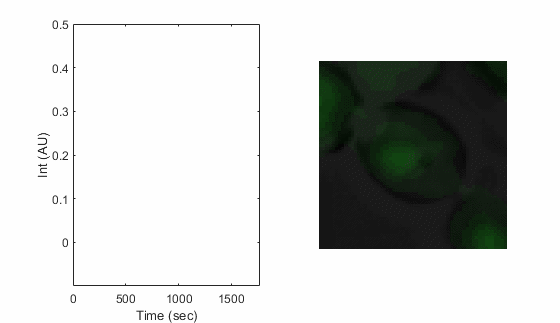

Supplement: S1 Movie — (GIF) [file pgen.1009629.s015.gif]

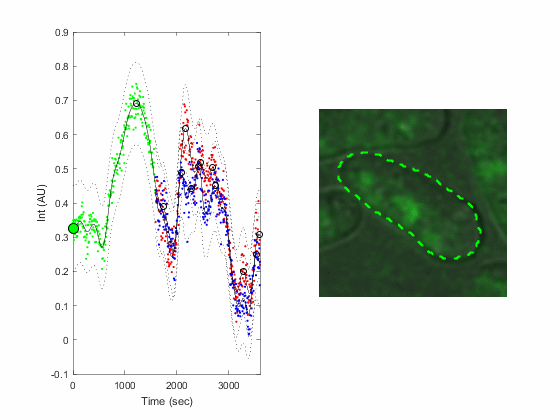

Supplement: S2 Movie — (GIF) [file pgen.1009629.s016.gif]
