# Supplementary material for: A functionally divergent intrinsically disordered region underlying the conservation of stochastic signaling
Source: PLoS Genet. 2021 Sep 10;17(9):e1009629. doi: 10.1371/journal.pgen.1009629 (PMC8457507; doi:10.1371/journal.pgen.1009629)
Supplement: S1 Text — (DOCX) [file pgen.1009629.s012.docx]

**Appendix**

Details of MI estimation for one experimental replicate

1. For each single cell
   1. Labeling calcium burst:
      1. Fit the trajectory of calcium reporter with the Gaussian Process described above to estimate basal fluctuations
      2. From the same trajectory, identify the highest 100 peaks (local maxima) with a minimal interval of 30 sec, and classify peaks into calcium bursts or basal fluctuations with the 95% CI of baseline estimated via the Gaussian Process, $\mathbf{Y}=\left\{ {y'}_{1},{y'}_{2},\ldots,{y'}_{100} \right\}^{T}, y_{i} \epsilon\left\{ {c^{'}}_{0},{c^{'}}_{1} \right\}$. Record the time points of each peak,$T=\left\{ t_{1},t_{2},\ldots,t_{100} \right\}, t_{i} \epsilon\left\{ 1,2,\ldots,p \right\}$. We used MATLAB function findpeaks to identify peaks and time points.
   2. Process pulsing dynamics of the *j*th block:
      1. Fit the trajectory of pulsing reporters with the Gaussian Process described above to smooth the trajectory
      2. For every calcium peak ${y'}_{i}$ the *j*th block was chosen, $X_{j}=\{x_{t_{i}+1+\tau(j-1)},x_{t_{i}+2+\tau(j-1)},\ldots,x_{t_{i}+\tau j}\}$. We arbitrarily chose $\tau=5$. Calculate $\mathbf{X}=\left\{ \mathbf{x}_{1},\mathbf{x}_{2},\ldots,\mathbf{x}_{100} \right\}^{T}, \mathbf{x}_{i}\boldsymbol{=}\left\{ \left\langle X_{j} \right\rangle_{i},\left\langle\dot{X_{j}} \right\rangle_{i} \right\}.$ $\mathbf{X}$ was jittered to avoid identical samples.
2. Concatenate every $\mathbf{X}$ and $\mathbf{Y}$ of the cell population. Randomly withdraw an equal number of samples for each burst label and reorganize the data into $D_{j}= \{\left( \mathbf{x}_{1j},{y'}_{1} \right),\left( \mathbf{x}_{2j},{y'}_{2} \right),\ldots,\left( \mathbf{x}_{nj},{y'}_{n} \right)\}$. Estimate $\mathrm{MI}_{j}$ by bootstrapping 600 times and report mean $\mathrm{MI}_{j}(\mathbf{x}_{j},y')$.

List of 40 fungi used for sequence analysis

| Candida dubliniensis |
| --- |
| Candida albicans |
| Candida tropicalis |
| Candida parapsilosis |
| Candida orthopsilosis |
| Lodderomyces elongisporus |
| Spathaspora passalidarum |
| Millerozyma farinose |
| Scheffersomyces stipites |
| Meyerozyma guilliermondii |
| Debaryomyces hansenii |
| Debaryomyces fabryi |
| Clavispora lusitaniae |
| Candida auris |
| Metschnikowia bicuspidata |
| Babjeviella inositovora |
| Saccharomyces cerevisiae |
| Saccharomyces mikatae |
| *Saccharomyces kudriavzevii* |
| *Saccharomyces uvarum* |
| Candida glabrata |
| Kazachstania Africana |
| Kazachstania naganishii |
| Naumovozyma castellii |
| Naumovozyma dairenensis |
| Vanderwaltozyma polyspora |
| Tetrapisispora phaffii |
| Tetrapisispora blattae |
| Zygosaccharomyces rouxii |
| Zygosaccharomyces parabialii |
| Torulaspora delbrueckii |
| Kluyveromyces lactis |
| Eremothecium gossypii |
| Achbya aceri |
| Eremothecium cymbalariae |
| *Lachancea kluyveri* |
| Lachancea thermotolerans |
| Lachancea waltii |
| Cyberlindnera fabianii |
| Wickerhamomyce ciferrii |
