# Supplementary material for: A functionally divergent intrinsically disordered region underlying the conservation of stochastic signaling
Source: PLoS Genet. 2021 Sep 10;17(9):e1009629. doi: 10.1371/journal.pgen.1009629 (PMC8457507; doi:10.1371/journal.pgen.1009629)
Supplement: S1 Table — (DOCX) [file pgen.1009629.s013.docx]

**S1_Table:**

| Plasmid ID | Description |
| --- | --- |
| pLC963 | pV1393-1 (CaCas9/sgRNA entry expression vector, contains NatR gene, targeting *NEUT5L*) [67] |
| pLC389 | GFP-NAT [68] |
