# Supplementary material for: A functionally divergent intrinsically disordered region underlying the conservation of stochastic signaling
Source: PLoS Genet. 2021 Sep 10;17(9):e1009629. doi: 10.1371/journal.pgen.1009629 (PMC8457507; doi:10.1371/journal.pgen.1009629)
Supplement: S2 Table — (DOCX) [file pgen.1009629.s014.docx]

**S2 Table:**

| oLC9367 | GATTATTATTAGAAGAACAAGCAAATTCAAGTGGTAAACCACTTGCTACTAGTAGTGGTGTTGAAATTACTGGTGGTGGTTCTAAAGGTGAAGAATTAT |
| --- | --- |
| oLC9368 | TGTGTGTGCGTGTGTGTGTAAATACTAAACCATTCGTATTTTTTTTTTTTTGCATCAAAATATACACATCGTAAAACGACGGCCAGTGAATTC |
| oLC9369 | ATTTTCAAACTGAAGCAGGG |
| oLC9370 | AATAAGTTTTGTTCCGGCAC |
| oLC9371 | CCCAGTTGCAATATGATAATcaaattaaaaatagtttacgcaagtc |
| oLC9372 | ATTATCATATTGCAACTGGGGTTTTAGAGCTAGAAATAGCAAGTTAAAA |
| oLC9373 | CACGATAGTTATTCCTGTTG |
